# Tomato Cultivars Resistant or Susceptible to Spider Mites Differ in Their Biosynthesis and Metabolic Profile of the Monoterpenoid Pathway

**DOI:** 10.3389/fpls.2021.630155

**Published:** 2021-02-26

**Authors:** Nati Weinblum, Alon Cna'ani, Beery Yaakov, Adi Sadeh, Lior Avraham, Itai Opatovsky, Vered Tzin

**Affiliations:** ^1^The Albert Katz International School for Desert Studies, Jacob Blaustein Institutes for Desert Research, Ben-Gurion University of the Negev, Beersheba, Israel; ^2^Jacob Blaustein Center for Scientific Cooperation, Jacob Blaustein Institutes for Desert Research, Ben-Gurion University of the Negev, Beersheba, Israel; ^3^French Associates Institute for Agriculture and Biotechnology of Drylands, Jacob Blaustein Institutes for Desert Research, Ben-Gurion University of the Negev, Beersheba, Israel; ^4^Southern R&D MOP-Darom, Negev, Israel; ^5^Agriculture Extension Service, Ministry of Agriculture and Rural Development, Bet Dagan, Israel

**Keywords:** *Solanum lycopersicum*, *Tetranychus urticae* (Koch), salicylic acid, volatile organic compounds, Terpene synthase, *Phytoseiulus persimilis*

## Abstract

The two-spotted spider mite (TSSM; *Tetranychus urticae*) is a ubiquitous polyphagous arthropod pest that has a major economic impact on the tomato (*Solanum lycopersicum*) industry. Tomato plants have evolved broad defense mechanisms regulated by the expression of defense genes, phytohormones, and secondary metabolites present constitutively and/or induced upon infestation. Although tomato defense mechanisms have been studied for more than three decades, only a few studies have compared domesticated cultivars' natural mite resistance at the molecular level. The main goal of our research was to reveal the molecular differences between two tomato cultivars with similar physical (trichome morphology and density) and agronomic traits (fruit size, shape, color, cluster architecture), but with contrasting TSSM susceptibility. A net house experiment indicated a mite-resistance difference between the cultivars, and a climate-controlled performance and oviposition bioassay supported these findings. A transcriptome analysis of the two cultivars after 3 days of TSSM infestation, revealed changes in the genes associated with primary and secondary metabolism, including salicylic acid and volatile biosynthesis (volatile benzenoid ester and monoterpenes). The *Terpene synthase* genes, *TPS5, TPS7*, and *TPS19/20*, encoding enzymes that synthesize the monoterpenes linalool, β-myrcene, limonene, and β-phellandrene were highly expressed in the resistant cultivar. The volatile profile of these cultivars upon mite infestation for 1, 3, 5, and 7 days, revealed substantial differences in monoterpenoid and phenylpropanoid volatiles, results consistent with the transcriptomic data. Comparing the metabolic changes that occurred in each cultivar and upon mite-infestation indicated that monoterpenes are the main metabolites that differ between cultivars (constitutive levels), while only minor changes occurred upon TSSM attack. To test the effect of these volatile variations on mites, we subjected both the TSSM and its corresponding predator, *Phytoseiulus persimilis*, to an olfactory choice bioassay. The predator mites were only significantly attracted to the TSSM pre-infested resistant cultivar and not to the susceptible cultivar, while the TSSM itself showed no preference. Overall, our findings revealed the contribution of constitutive and inducible levels of volatiles on mite performance. This study highlights monoterpenoids' function in plant resistance to pests and may inform the development of new resistant tomato cultivars.

## Introduction

In response to herbivore attack, plants express broad phenotypic plasticity to defend themselves. These defense strategies directly and/or indirectly affect herbivores and combine pre-existing, developmentally regulated constitutive defenses with inducible processes modified in response to specific herbivores (Santamaria et al., 2020). Direct mechanisms include morphological structures, such as hairs, trichomes, and the production of compounds such as latex, acyl-sugars, waxes, and callose that form the first barrier discouraging herbivore attack (Santamaria et al., 2013). Plants also synthesize defensive proteins and small molecules (i.e., secondary metabolites) consumed by herbivores, which repel or toxify them and reduce their fitness (Fürstenberg-Hägg et al., 2013). Moreover, they produce volatile organic compounds (VOCs) involved in direct and indirect defense responses (War et al., 2012). Ubiquitous and species-specific plant volatiles belong to a wide range of different biochemical classes, mainly phenylpropanoids and benzenoids, terpenoids, and aliphatics, which play a key role in direct or indirect defense (Cheynier et al., 2013; Kessler, 2017; Ameye et al., 2018).

Complex blends of volatiles released into the atmosphere can mediate both direct defense, by repelling herbivores, and indirect defense, by attracting natural enemies such as predators and parasitoids (Kessler and Baldwin, 2001), deterring oviposition (De Moraes et al., 2001), and even priming neighboring plants and distal plant parts (Frost et al., 2008; Heil, 2014; Agut et al., 2015). These molecules can be emitted either continuously or as herbivore-induced plant volatiles (HIPVs). The largest and most diverse class of plant volatiles functioning in plant defense are terpenoids (Aharoni et al., 2006), classified into hemiterpenes, monoterpenes, sesquiterpenes, and diterpenes. Another category of VOC terpenoids includes those with irregular structures, for example, homoterpene 4,8,12-trimethyltrideca-1,3,7,11-tetraene (TMTT) (Ament et al., 2006), and β-ionone, the catabolism product of tetraterpenes (Cáceres et al., 2016). VOCs produced by tomato plants are predominantly monoterpenes, and a variety of sesquiterpenes (Markus Lange and Ahkami, 2013). Some monoterpenes display pesticidal activity (deterring oviposition and digestive processes) against different arthropods (Keeling and Bohlmann, 2006), including mites (Agut et al., 2014), and some exhibit repellent properties (De Moraes et al., 2001), while sesquiterpenes are involved in indirect responses.

An effective defense against pests depends not only on secondary metabolite accumulation but also on hormonal signaling. This process is mediated by several phytohormone signaling pathways, mainly salicylic acid (SA), jasmonic acid (JA), abscisic acid (ABA), and ethylene (ET) (Pérez-Hedo et al., 2018). These phytohormones activate different signaling cascades that regulate transcriptional responses, followed by the downstream synthesis of secondary metabolites, protease inhibitors, and other defenses that have toxic, repellent, and/or anti-nutritive effects on herbivores (Takabayashi et al., 2000). The transcriptomic and metabolomic responses to the feeding of the two-spotted spider mite (TSSM; *Tetranychus urticae* Koch) have been studied in multiple plant species, including Arabidopsis (Zhurov et al., 2014), tomato (Martel et al., 2015), pepper (*Capsicum annuum*) (Zhang et al., 2020), and cucumber (*Cucumis sativus*) (He et al., 2020), and the results have suggested that JA is the principal phytohormone regulating the induction of plant defenses against herbivores (Zhurov et al., 2014; Rioja et al., 2017). SA and its methylated volatile form MeSA also play an important role in determining TSSM response intensity and the crosstalk between SA and JA (Thaler et al., 2012). In the tomato, in some cases, the TSSM triggers the expression of genes encoding for the biosynthesis of both JA and SA (Ament et al., 2004; Martel et al., 2015), while in the lima bean (*Phaseolus lunatus*), the two phytohormones have shown an antagonistic relationship, modulating indirect volatile emission (Wei et al., 2014). These results indicate that the phytohormone crosstalk is species-specific and can vary between different cues.

The TSSM is an important polyphagous arthropod herbivore, feeding on greenhouse, field, and orchard crops worldwide (Weintraub and Palevsky, 2008). It was reported to infest over 1,100 plant species, including more than 150 crops (Martel et al., 2015), especially within the *Solanaceae* family (Migeon et al., 2009). The TSSM's wide host range, short life cycle, and straightforward maintenance in the laboratory, combined with its genomic and genetic tools, make it an attractive model pest for elucidating the molecular mechanisms of plant-herbivore interactions and plant defense mechanisms (Zhurov et al., 2014; Rioja et al., 2017). TSSMs feed through their mouthpart stylet adapted for a sucking mode of feeding on the cells within the leaf mesophyll (Park and Lee, 2002). They reach the cells with their stylet, either through stomatal openings or in between the intercellular space of the epidermal cells without damaging them (Reddall et al., 2004; Bensoussan et al., 2016). Due to their small body size and short life cycle, TSSMs often remain unnoticed until their presence is revealed by plant damage. Hence, their control mostly depends on the application of synthetic insecticides and acaricides (Bolland et al., 1998). However, TSSM populations can develop resistance toward these compounds, and their control has become problematic in many regions in the world (Van Leeuwen et al., 2010). A predator commonly used commercially as a biological control is *Phytoseiulus persimilis*, which feeds on all TSSM life stages (Khalequzzaman et al., 1970). *Phytoseiulus persimilis* mites are blind and use olfactory cues to locate their prey (Van Den Boom et al., 2004; Kappers et al., 2011). Upon TSSM infestation, tomato plants emit volatiles as a signal to attract the predatory mite (Takabayashi et al., 2000). The application of *P. persimilis* on tomato plants has not been widely adopted, probably due to its inconsistent performance (Escudero and Ferragut, 2005), the requirement of intense monitoring of the prey population, and variation throughout plant growth. Thus, a combination of mild acaricides and biological control approaches has been recommended for farmers, in addition to improving genetic variation for mite resistance.

The tomato (*Solanum lycopersicum* L.) is one of the most popular and economically valuable vegetables worldwide. Elite cultivars suffer from severe yield loss due to susceptibility to diseases caused by all types of pathogens (i.e., viruses, bacteria, and fungi) and pests such as nematodes, insects, and mites. This susceptibility is due to the strong genetic bottlenecks introduced during domestication and modern breeding (Bai and Lindhout, 2007). Therefore, improved resistance is a desirable trait. Consequently, the available cultivated tomato varieties present a large diversity of fruit-related traits and adaptation to different habitats (Schauer et al., 2005), but they show reduced resistance to pests, or lack it altogether (Escobar-Bravo et al., 2016). Wild tomato genotypes evolved both physical and chemical barriers as resistance mechanisms against the TSSMs, including synthesizing acyl-sugars, methyl ketones, and terpenoids, accumulated in the trichomes, while in cultivated tomatoes, these mechanisms are reduced or completely lost (Escobar-Bravo et al., 2016; Rioja et al., 2017). Resistance to TSSMs could be achieved by improving constitutive and/or inducible levels of physical and/or chemical defenses. A few studies successfully improved TSSM resistance by transferring trichome-based compounds, high in acyl-sugars, from wild tomatoes into susceptible cultivated tomatoes (Fernández-Muñoz et al., 2000; Rakha et al., 2017). However, these data have not yet been translated into commercial cultivars. Other studies have highlighted the importance of the genotype-based volatile composition of tomatoes and indicated its potential role in the interaction between the host plants, pests, and natural enemies (Keskin and Kumral, 2015). It is unknown which volatiles affect TSSM recognition and attraction to tomato plants or affect TSSM performance on the plant. The effects of mite-induced plant volatiles on natural enemies have been previously studied. Nevertheless, to our knowledge, no study has compared the volatile and transcriptional changes of commercial tomato cultivars and their effect on TSSMs.

In this research, we investigated the constitutive and inducible molecular levels that cause variations in mite susceptibility. We assessed the relative TSSM susceptibility of two cluster cherry tomato cultivars with similar fruit shape, size, and markets, in net house conditions, and determined the mite population size and their damage to plants. Second, the performance and oviposition of TSSMs were evaluated on whole plants and intact tomato leaves, respectively. Third, we used gene expression analysis to assess which biosynthetic and signaling pathways may be involved in these plant defenses, followed by a time point analysis of volatiles. Finally, an olfactory analysis of TSSMs and *P. persimilis* revealed different volatile blends emitted by plants under naïve or infested conditions. Three *TPS* genes were found to have potential value as TSSM resistance genes for breeding new resistant varieties. The results obtained in this study provide useful data for improving volatile content in the tomato, which can be adjusted to constitute a comprehensive pest management program for the TSSM in tomato fields and net houses.

## Materials and Methods

### Evaluation of TSSM Populations and Plant Damage

Two commercial cluster cherry tomato cultivars were selected: Ofir (Rimi Ltd., Israel) and Shiran (Hazera Seeds Ltd., Israel). According to the Rimi website (https://www.rimi.co.il/; in Hebrew), Ofir was reported as a mite-resistant cultivar relative to the other cultivars. Seeds were germinated at a nursery (Hishtil Ltd., Israel), and 1-month-old seedlings were transplanted directly in the ground in net houses located at the R&D Southern Station (MOP Besor). From each cultivar, 45 plants were planted under a net house condition (Supplementary Figure 1). The net house size is 6 m^2^ and is entirely covered by a white 50-Mesh net, which is used for blocking pests such as whiteflies, aphids, and leafminers. Plants were grown for 3 months with an irrigation system and were treated with pesticides except for acaricides. From the 2nd week after transplanting, the mite population was monitored (usually once a week). For mite infestations, tomato leaves with dense TSSM populations were intentionally introduced to the net house and spread throughout the central rows (Supplementary Figure 1 middle rows, #3 and #4). Mites were counted from 9 leaves per plant (three from each position, top, middle, and bottom) of the 20 plants in rows #2 (Shiran), and #5 (Ofir). The mites were scored according to two phenologies: juvenile and adult, along with a total of 9 sampling time points. Plant damage was evaluated once, at 5 weeks after TSSM infestation, divided into three categories: (i) low, (ii) mild, and (iii) severe damage.

### Growing Plants Under Controlled Growth Conditions and Mite Rearing

Tomato seeds of the two commercial tomato cultivars, Ofir and Shiran were sown in plastic pots, each containing ~500 cm^3^ of a tuff mixture with vermiculite (2:1) and an N-P-K fertilizer (20–20–20). Plants were maintained under controlled growth conditions (16 h/8 h light/dark; 250–350 μmol photon m-2 s-1 light intensity from a 3,000 lm LED; 60–70% RH; 24 ± 2°C), watered every day, and fertilized twice a week with a balanced nutrient solution. Plants used in the experiments were 4–5 weeks old. The two-spotted spider mite (TSSM), *Tetranychus urticae*, and the predatory mite, *Phytoseiulus persimilis*, were obtained from Biobee Sde Eliyahu Ltd. They were reared in a climate-controlled room (24 ± 2°C, 60 ± 10% RH, 16 h/8 h light/dark). The TSSMs were maintained on tomato plants, and the predatory mites were reared on detached tomato leaves infested with TSSMs on wet cotton wool inside plastic trays.

### TSSM Performance and Fecundity

Ofir and Shiran plants were grown in a climate-controlled room for 3-weeks, then infested with ten 4 to 5-day-old TSSM, which were randomly transferred to each plant with a fine camel paintbrush. Six tomato plants of each cultivar were used per assay, which was repeated three times. To prevent mite's movement from one plant to another, plants were isolated using a plastic tray filled with water. Samplings were conducted 13 days after mite infestation (dpi), and leaves were inspected visually to count the total number of TSSMs on each plant. To evaluate oviposition, 10 leaf disks (2 cm in diameter) of tomato leaves from each of the two cultivars were placed upside down on water-saturated cotton wool in a plastic container (20 cm×8 cm×3 cm); 6 sets of these containers were prepared, for a total of 30 leaf-disks per cultivar. One female spider mite (4–5 days old) was transferred using a fine brush, onto each of the cut leaf disks and allowed to oviposit under laboratory conditions (24 ± 2°C, 60 ± 10% RH, L16:D8). The eggs laid by each individual female were counted daily under a binocular microscope (10–20×) for 5 days or until the female died.

### RNA Extraction, Libraries, and Sequencing

Leaflets from 5-weeks old tomato plants were either infested with 15 TSSMs on four different leaflets (a total of 60 mites per plant) for 3 days (sample name Oi-3d and Si-3d) or remained untreated as a control (sample name Oc-3d, and Sc-3d). Then, leaflets were harvested, and two leaflets were pooled for each biological replicate and immediately frozen in liquid nitrogen. Sampling included three replicates for each treatment, except for Shiran control treatment that had only two replicates sampled on the 3rd day. Therefore, we extracted two additional Shiran control samples from the same experiment, collected on the 1st day (sample name Sc-1d). Total RNA was extracted using an SV Total RNA Isolation Kit with an on-column DNaseI treatment (QIAGEN), quantified, and 2.5 μg of each sample was dried in RNA protective tubes (GenTegra LLC, USA). After the library preparation, paired-end (150 bp read length) RNA sequencing was conducted using an Illumina HiSeq 4000 instrument performed by the GeneWIZ Company (www.genewiz.com). Quality control was performed using FASTQC, where low-quality sequences and adapters were trimmed and excluded using Trimmomatic v0.36.

### Transcriptome Analysis

Mapping was performed using a STAR aligner v2.5.3a against the *Solanum lycopersicum* genome (SL3.0; EnsemblPlants release 47) reference transcriptome (ftp.ensemblgenomes.org/pub/plants/release-47) using sjdbOverhang of 149 and quantMode of “geneCounts.” The mapped sequence reads showed a high percentage of uniquely mapped reads of the two cultivars (Supplementary Table 1). Gene counts were transformed to trimmed mean of M-values (TMM) (Robinson and Oshlack, 2010) using the bcbioRNAseq v0.3.39 (Supplementary Table 2). DESeq2 v1.26.1 (Love et al., 2014) was used for differential gene analysis [adjusted *p* < 0.05; |log_2_ (fold change)| ≥1] with design ~Infestation + Cultivar and contrasting Shiran vs. Ofir (reference variable) and Infested vs. Control (reference variable). The data for the transcriptomic overview PCA were transformed using a regularized log (rlog) transformation. A functional ontology enrichment analysis was performed using topGO v2.42.0 (Alexa et al., 2006) with Fisher's exact test. A comparison between the TMM values of genes from two selected pathways, terpenoid and salicylic acid biosynthetic genes, showed a high similarity between the 11 samples (only 3d) and the 13 samples (3d and 1d) as shown in Supplementary Figure 2. The TMM datasets are presented in Supplementary Table 3. Due to the high similarity, we decided to conduct Student's *t*-test analyses, including the two additional samples of Shiran control 1d, and to present the data in fold change. The raw sequence data have been submitted to the NCBI Sequence Read Archive (SRA) accession PRJNA663461.

### Volatile Profiling Using Headspace Solid-Phase Micro-Extraction Coupled With Gas Chromatography-Mass Spectrometry (HS-SPME-GC-MS)

Ofir and Shiran plants were grown in a climate-controlled room for 5 weeks, then infested with 15 TSSMs on four different leaflets, for a total of 60 mites per plant. Samples were collected at multiple time points 1, 3, 5, and 7 dpi. Leaflets (1 gr) were collected in 15 ml falcon tubes, immediately frozen in liquid nitrogen, and stored at −80°C. For each time point and cultivar, 5 biological replicates were collected, including untreated leaflets (at each time point). Then, tissue was ground in liquid nitrogen and placed in 20 ml glass vials (CleanVial, Chrom4, Thüringen, Germany), which also contained 1 gr of NaCl and 7 ml of a 20% (w/v) NaCl solution. Additionally, *Iso*butylbenzene (10 mg/L, Sigma-Aldrich, Israel), was added to each vial as an internal standard. The volatile profiles were examined by headspace solid-phase microextraction (HS-SPME) coupled with GC-MS. Prior to analysis, glass vials were incubated for 15 min at 60°C with PAL COMBI-xt (CTC Analytics AG Switzerland) to release free volatiles into the headspace. A 10 mm long SPME fiber, assembly 50/30 μm, divinylbenzene/carboxen/polydimethylsiloxane (Supelco, Bellefonte, PA, USA), was introduced into the headspace for 15 min at 60°C. The fiber was then desorbed for 10 min at 250°C in splitless mode within the inlet of a 7890A GC (Agilent, Santa Clara, CA, USA) equipped with an VF-5MS 10 m EZ guard capillary column (30 m × 0.25 mm inner diameter, 0.25 μm film thickness; Agilent CP9013, USA), coupled to a 5977B MS detector (Agilent). Helium was the carrier gas in a constant pressure mode rate of 1 mL·min^−1^, and the GC temperature was programmed for 40°C (1 min), and increased to 250°C at 6°C /min. Ionization energy was 70 eV with a mass acquisition range of 40–400 *m/z*, and a scanning rate of 6.34 spectra/s. Retention index (RI) was calculated by running C8–C20 *n*-alkanes. Compounds were identified by Wiley 10 with NIST 2014 mass spectral library data using the Mass Hunter software package (version B.08.00, Agilent, USA). Further identification of major compounds was based on a comparison of mass spectra and the retention index. Compounds with authentic standards (Sigma-Aldrich, Israel) were analyzed under similar conditions. Quantitative evaluation was performed using internal standard; peak areas were normalized that of to the *Iso*butylbenzene, Sigma-Aldrich, Israel, 0.8 μg per sample. Retention indices of the compounds and the reference source can be found in Supplementary Table 6. The experiment was repeated twice for all time points and repeated three times for 1 and 3 dpi.

### Volatile Profile Measurement Using a Gas Chromatography-Mass Spectrometry (GC-MS) Liquid Extraction

Leaflets from the net house experiment were sampled eight times during along the growth season (from August till October 2017). To determine the internal pool compositions of volatile compounds in tomato plants, leaflets (1 gr from three leaflets per sample, five replicates for each treatment) were harvested periodically over the season and immediately flash-frozen in liquid nitrogen. Samples were ground in liquid nitrogen, then extracted in hexane (4 ml gr^−1^ tissue) supplemented with 4 μg of *Iso*butylbenzene (Sigma Aldrich, Israel) as the internal standard. Following a 2 h incubation with shaking at 200 rpm, samples were centrifuged at 10,000 g for 10 min. Supernatants were then removed and flush-concentrated ~40-fold under the nitrogen stream prior to chromatography. For GC-MS analysis, 1 μL of the sample was injected by a HT2800T autosampler (HTA, Italy), into a TRACE GC ULTRA gas chromatograph (Thermo-Fisher, USA) as previously described (Kumari et al., 2020). The compound identification as described above.

### Two-Choice Bioassay Using Y-Shape Olfactometer

Mite responses to volatiles were observed by a two-choice vertical olfactometer previously described by Pallini et al. (1997) and Gyan et al. (2020) with minor adjustments. A Y-shape glass tube (3.5 cm inside diameter) was formed with a base arm (20 cm in length) and two side arms of 15 cm in length at 75° angle, with a Y-shaped metal wire (1 mm thick) in the middle to channel the mites. The two side arms were each connected with polytetrafluoroethylene (PTFE) tubing to a glass beaker (10 L in volume) containing one potted tomato plant, serving as an odor source. The plant was placed in a small tray inside a second tray containing water, which served as an airtight seal for the glass beaker. Airflow was provided by an aquarium air compressor pump—pushing air from the odor source to the side arms of the Y-tube, adjusted with a flow meter to 0.4 L × min^−1^ for each arm. Before reaching the two glass beakers, the air passed through an activated charcoal filter (Millennium, HI, USA). Individual mites were placed on the wire in the base of the Y-tube with a paintbrush after being starved for at least 2 h. Each mite was observed until it moved at least 10 cm through one of the side arms. Mites that did not choose a sidearm within 5 min, were considered as having made no-choice and were excluded. Each female was tested only once in the Y-tube selection system. For each pair of volatile sources (plants), 40 adults were tested on two different experimental days. To minimize positional bias, after testing a batch of five females, the volatile sources were switched between the sides of the arms. After testing 10 females, the Y-tube and glass beakers were washed with ethanol (70%). To eliminate the possible effect of light source, a 20 W fluorescent light was placed in front of the two side arms of the Y-tube. Mite bioassays were carried out in a climate-controlled room at 24 ± 2°C, 60 ± 10% RH. Mite responses were assessed for combinations of the following treatments: (i) mite-resistant tomato cultivar, free of TSSMs (Ofir-control), (ii) mite-susceptible tomato cultivar, free of TSSMs (Shiran-control), (iii) mite-resistant tomato cultivars, infested with TSSMs (Ofir-infested), and (iv) mite-susceptible tomato cultivar, infested with TSSMs (Shiran-infested). For the infested treatment, plants were infested with 60 spider mites for 3 days prior to the experiments.

### Statistics

For the RNA-seq principal component analysis (PCA) whole transcriptomic overview, 22,592 genes were selected (transcripts with only zero values were excluded). Then, regularized log-transformed data were scaled to the average and standard deviation [(x – x)/σ], calculated and designed using R. For the PCA of selected genes (SA and terpenoid biosynthesis), the missing values (zeros) were replaced by LoDs (1/5 of the minimum positive value of each variable), normalized with a log transformation using MetaboAnalyst tool (Xia et al., 2009). Data for the PCA plot of the VOCs were log-transformed and calculated using MetaboAnalyst tool. The Venn diagram was designed using the Venny 2.1.0 drawing tool (http://bioinfogp.cnb.csic.es/) using DEGs data. The oviposition results were analyzed by two-way ANOVA (analysis of variance), and one-way ANOVA (each time point or leaf section), followed by a *post-hoc* test using Tukey's HSD. For gene pair-wise comparisons, a Student's *t-*test was used, *p-*value adjusted (FDR; false discovery rate), and data were presented in log2 fold change. For the two-choice vertical olfactometer bioassay, a Chi-square (χ^2^) goodness of fit test based on a null model, was used, where the odor sources were selected with equal frequency (50:50 response). These analyses were conducted using JMP13 software (SAS; www.jmp.com) and Microsoft Excel.

## Results

### Evaluation of Mite Populations in the Net House and Their Damage to Tomato Leaves

**Figure 1:**
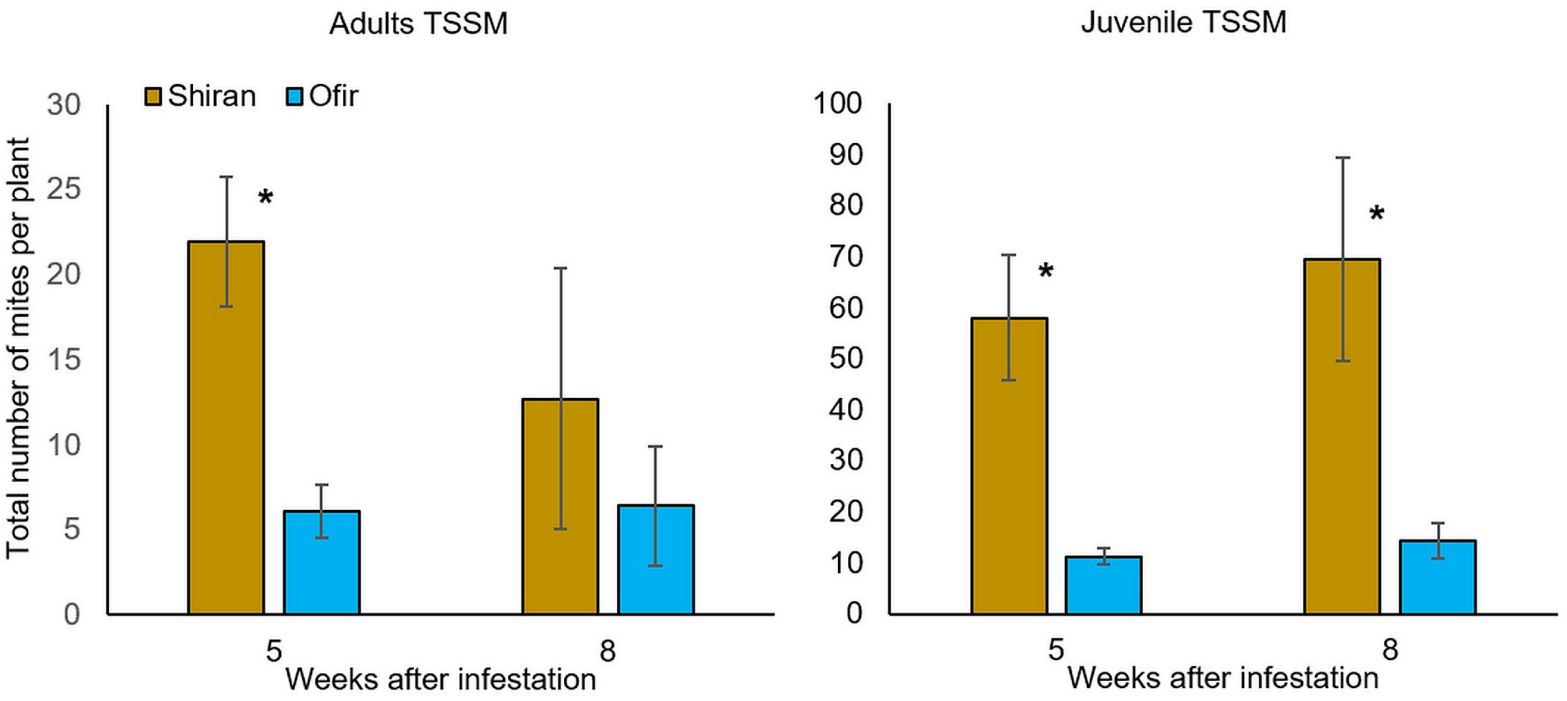
The total number of adult and juvenile TSSMs per plant on the two tomato cultivars grown in a net house. Mite population was counted 5 and 8 weeks after TSSM infestation. At each time point, nine leaves from a plant were selected, twenty plants of each cultivar were sampled (Student's *t*-test; *p < 0.05; *n* = 20; mean ± SE). The time points that are presented are the ones in which the peak of the mite population was observed.

The two commercial tomato cultivars (Shiran and Ofir) were selected based on the following information: description on the Rimi website (www.rimi.co.il), and pre-observation by the Agriculture Extension Service of Israel that reported a lower mite population on Ofir than on the other commonly grown cultivars of cluster cherry tomato. Mite populations were evaluated according to the adult and juvenile pest life cycle stages (eggs were excluded). The average numbers of adult and juvenile mites on two sampling dates, 5 and 8 weeks after TSSM infestation, are presented in Figure 1. A comparison of the two sampling dates indicated that Shiran had more mites than Ofir in the juvenile stages, while only on the 5 weeks the adult stage had significantly higher on Shiran than on Ofir. In the 8 weeks, the adult mite population on Shiran had reduced, and in Ofir, it was not changed, which might be the outcome of severe plant damage. Supplementary Figure 3 shows the total number of adult and juvenile mites on the two tomato cultivars along 2 months of scouting for mites. These results also show a higher number of both adults and juveniles on Shiran vs. Ofir. Overall, they suggest that in net house growth conditions, Shiran is more mite susceptible than Ofir.

**Figure 2:**
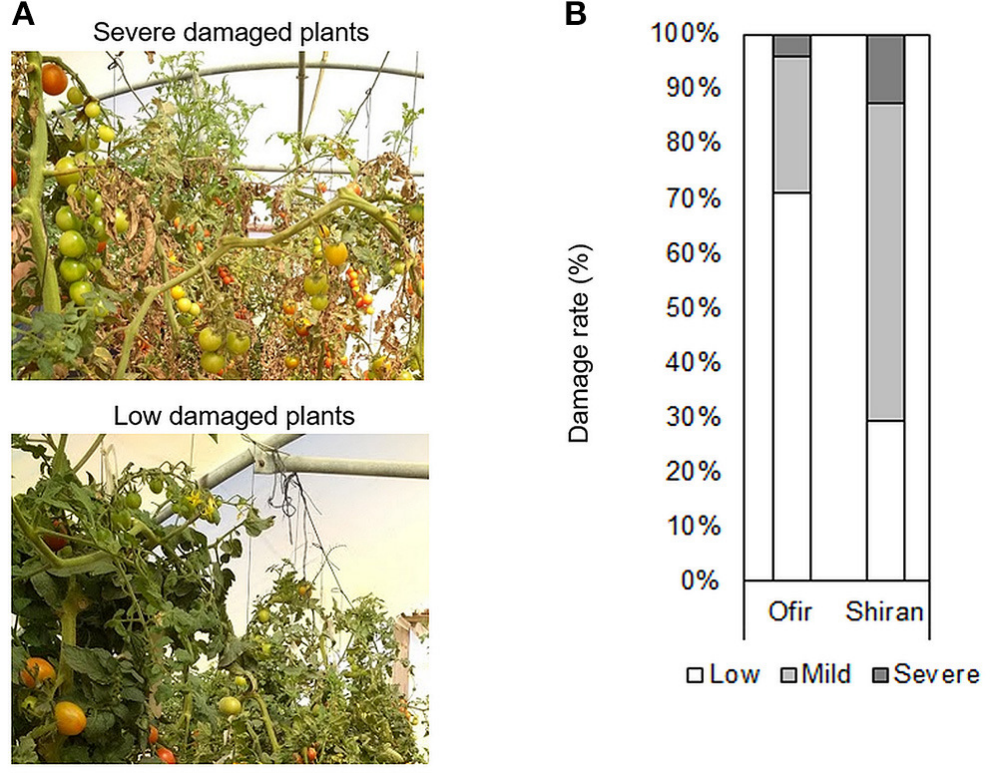
Plant damage rate of the tomato cultivars grown in a net house. **(A)** Photos from the tomato plants. **(B)** Plant damage rate of whole tomato plants. Plant severity symptoms were scored on three damage levels: (1) slight, (2) mild, and (3) severe. *n* = 24–26.

Additionally, plant morphology was scored at a single time point (5 weeks after TSSM infestation; Figure 2A), 3 months from the beginning of the experiment. Figure 2B presents the distribution of the plant damage in the two tomato cultivars in the net house. Most Shiran plants were mildly damaged and more damaged than most of the Ofir plants. These results suggest that the plant damage was related to the intensity of mite infestation, and that the Ofir cultivar is more resistant to mites.

### Evaluating TSSM Performance and Fecundity Under Controlled Growth Conditions

**Figure 3:**
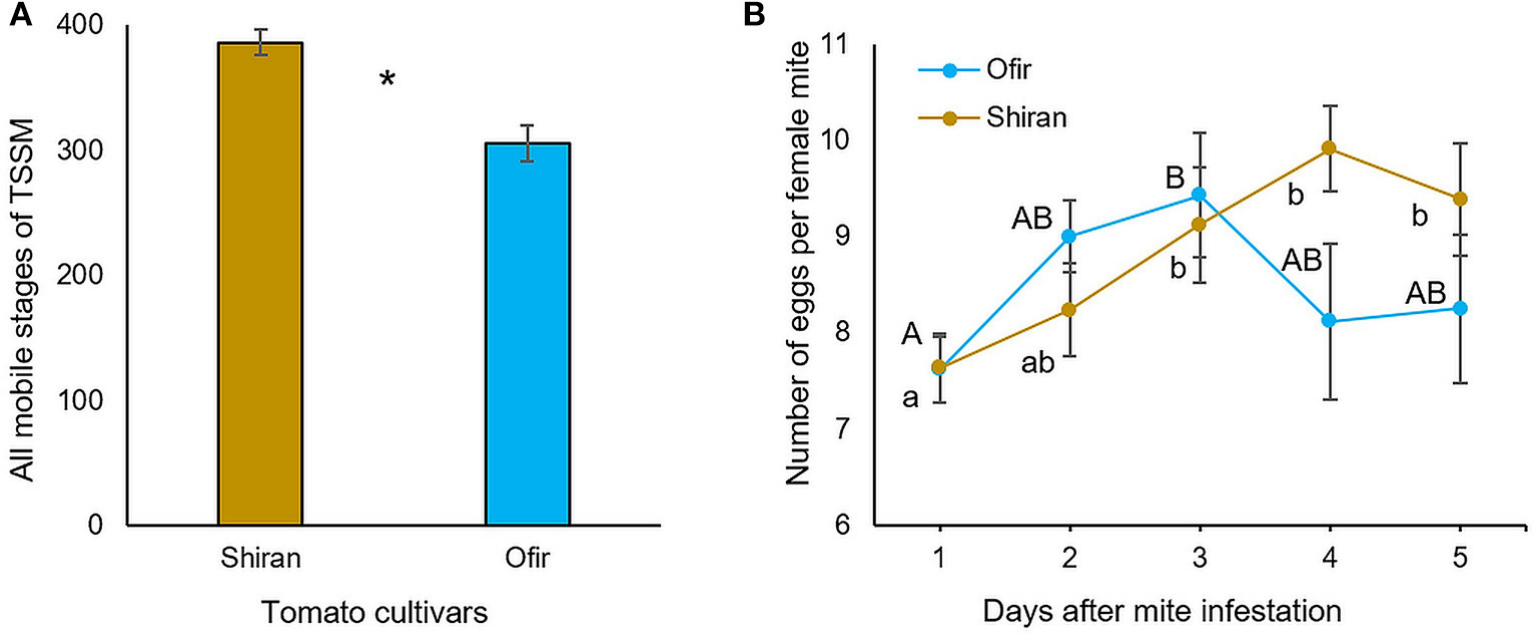
Evaluation of TSSM performance and oviposition rate on the two tomato cultivars Ofir and Shiran. **(A)** The mites were counted 13 days after infestation, 4-week-old tomato plants were infested with 10 TSSMs per plant. Asterisks indicate significant differences between cultivars (Student's *t*-test; *p < 0.01; *n* = 6; mean ± SE. **(B)** Oviposition rate was determined from 30 sets of leaf disks infested with one adult female mite per leaf disk. The figure shows the average total egg production per day. There was no significant difference between the number of eggs produced in the cultivars (ANOVA with *df* = 1; *F* = 1.1; *p*-value = 0.29). There was a significant difference in the number of eggs between sampling days in each cultivar (ANOVA with *df* = 4; *F* = 6.3; *p* < 0.0001). Different letters indicate significant differences determined by one-way ANOVA (*p* < 0.05; HSD).

To validate whether Ofir was more resistant to TSSMs than Shiran in a controlled environment, two complementary experiments were conducted: (i) mite reproduction, in which the number of mobile mites was evaluated on whole mite-infested plants for 13 days; and (ii) mite oviposition, in which the number of eggs was counted for 5 days during mite infestation using leaf disks. As shown in Figure 3A, mite reproduction was significantly higher on Shiran (386.5) than on Ofir (306.2) plants, suggesting that Shiran is more susceptible to TSSMs under laboratory conditions. We determined the oviposition rate of TSSMs on the cultivars by applying females and counting new eggs each day, as shown in Figure 3B. The two-way ANOVA indicated a significant difference in the number of eggs between sampling days (*df* = 4; *F* = 6.3; *p* < 0.0001), while no significant difference was detected between the cultivars (*df* = 1; *F* = 1.1; *p* = 0.29). Therefore, we conducted a one-way-ANOVA comparing the number of eggs on each cultivar separately. On Ofir, the highest number of eggs was counted on the 3rd day (9.4 eggs per female), then it declined to 8.3 eggs per female on the 5th day, while on Shiran, the highest numbers of eggs were counted on days 3–5 (9.1–9.9 eggs per female). These results revealed the different time responses of the two cultivars, possibly driven by variations in the molecular mechanisms.

### Pathway Analysis and Identification of Relevant Metabolic Pathways Mediating the Tomato Responses Against TSSMs

**Figure 4:**
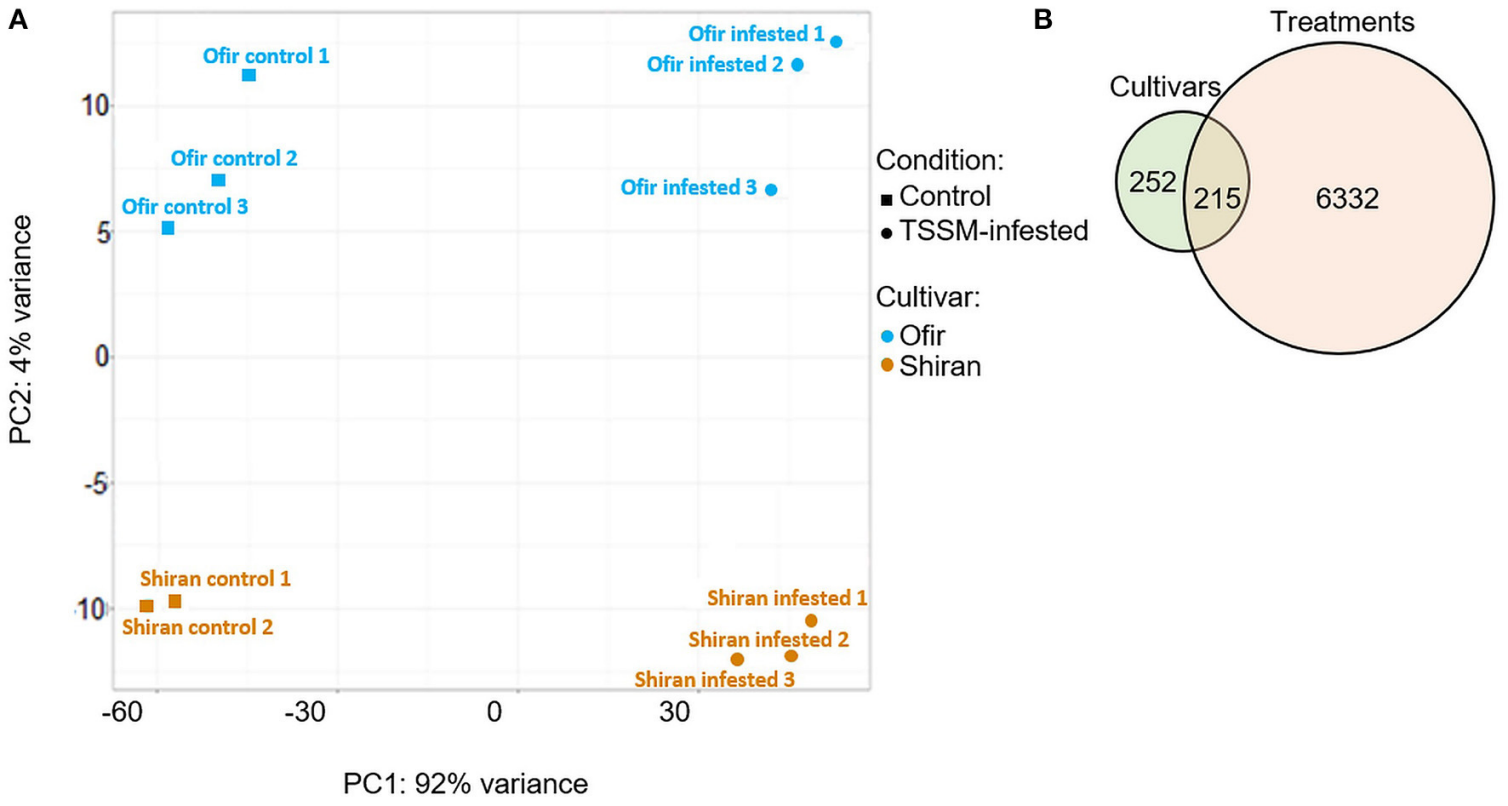
Transcriptomic overview of two tomato cultivars infested with TSSMs for 3 days. **(A)** PCA plot was generated using 22,592 genes (transcripts with only zero values were excluded). **(B)** Venn diagram illustrates the number of genes that were differentially expressed (DEGs) between the two cultivars (Shiran/Ofir) and/or treatments (TSSM-infested/control). Absolute fold change >|2|, *p* < 0.05 FDR (*n* = 5–6 biological replicates).

To investigate the global transcriptional changes of the tomato cultivars, leaves from 5-week-old plants were infested with 60 TSSMs for 3 days, and then samples from TSSM-infested and untreated controls were collected. The total transcript levels (22,592 genes with no zero values) were used to construct a principal component analysis (PCA) plot. As presented in Figure 4A, the PCA plot indicates that each cultivar's samples were clustered with one another. The samples were separated into infestation treatments, which explained 92% of the variance (PC1), and into cultivars, which explained 4% of the variance (PC2). This analysis suggests strong inducible responses of both cultivars following TSSM infestation, as well as distinct constitutively expressed sets of genes between the two cultivars. The DESeq2 tool was used to detect differentially expressed genes in the transcriptomic dataset (Love et al., 2014), which generated two sets of transcriptional variations: (i) between the two cultivars and (ii) between treatments (TSSM-infested vs. untreated control). A list of the gene sets is presented in Supplementary Table 3. The analysis of differentially expressed genes resulted in a total number of 7,099 differentially expressed genes, and the distribution of these genes was calculated and is presented in a Venn diagram (Figure 4B). Many genes were altered in response to mite attack (6,632), while only a small number of genes (252) were differentially expressed between the two cultivars. Additionally, 215 genes varied between both cultivar and treatment. This result indicates that the major transcriptomic effects are induced by TSSM infestation.

To reveal the biological processes involved in each group of the Venn diagram, an over-representation pathway enrichment analysis was performed, and the results were compared using the LycoCyc database (http://solcyc.solgenomics.net/). In this analysis, the differentially expressed genes were included (both up- and downregulated in each group). In Table 1, the significantly enriched pathways of cultivars, TSSM-induced treatment, and overlap group genes are presented (Tables 1A–C, respectively). The pathways significantly enriched by the cultivars alone were mainly associated with the biosynthesis of phenylpropanoids (flavonol, syringetin, luteolin, and leucopelargonidin and leucocyanidin) and terpenoids (monoterpenes). A large number of over-represented pathways were enriched upon mite infestation, including the following central metabolic pathways: amino acid biosynthesis (Phe, Tyr, Trp, Asp, and Glu), fatty acid and lipid biosynthesis (phospholipids), photosynthesis, and carbohydrate metabolism (Table 1B). Additionally, several pathways associated with secondary compound biosynthesis were enriched: cofactor, prosthetic group, electron carrier, and vitamin (folate and chlorophyll cycle), nitrogen-containing compounds (hydroxycinnamic acid tyramine amides), and terpenoid (antheraxanthin and violaxanthin, and phaseic acid). Notably, another over-represented pathway was the biosynthesis of volatile benzenoid esters (i.e., MeSA and methylbenzoate), which result from the degradation of aromatic compounds (Tzin and Galili, 2010).

**Table 1:** Pathway enrichment analysis of significantly altered gene expression between the two tomato cultivars and in response to TSSM infestation.

**Super pathway**	**Pathway name**	***P*-value**	**Number of genes**
**(A) CULTIVAR ONLY**			
Phenylpropanoids	Flavonol biosynthesis	1.29E-03	4
	Syringetin biosynthesis	2.34E-03	4
	Luteolin biosynthesis	9.04E-04	3
	Leucopelargonidin and leucocyanidin biosynthesis	1.81E-02	4
Terpenoid biosynthesis	Monoterpene biosynthesis	6.67E-04	3
**(B) TREATMENT ONLY**			
Amino acid biosynthesis	Superpathway of phenylalanine, tyrosine, and tryptophan biosynthesis	9.89E-03	23
	Tyrosine biosynthesis	3.17E-02	3
	Asparagine biosynthesis	3.89E-02	7
Amino acid degradation	Glutamate dependent acid resistance	1.58E-02	4
	Aspartate degradation	4.16E-02	9
Aromatic compound biosynthesis || Phenylpropanoids || Phytohormones	Volatile benzenoid ester biosynthesis	4.71E-03	5
Carbohydrate metabolism	Melibiose degradation	3.96E-03	4
Cofactor, prosthetic group, electron carrier, and vitamin biosynthesis	Folate transformations	1.41E-02	10
	Chlorophyll cycle	9.92E-04	5
Detoxification	Removal of superoxide radicals	1.26E-02	9
Fatty acid and lipid biosynthesis	Phospholipid desaturation	4.29E-03	6
	Phosphatidylcholine biosynthesis	3.17E-02	3
Nitrogen-containing secondary compound biosynthesis	Hydroxycinnamic acid tyramine amides biosynthesis	9.49E-03	9
Photosynthesis	Oxygenic photosynthesis	1.76E-04	32
Terpenoid biosynthesis	Antheraxanthin and violaxanthin biosynthesis	1.58E-02	3
	Phaseic acid biosynthesis	4.06E-02	5
**(C) OVERLAP (CULTIVARS AND TREATMENTS)**			
Phenylpropanoids	Phenylpropanoid biosynthesis, initial reactions	1.57E-03	3
	Suberin biosynthesis	4.72E-03	5
	Flavonol biosynthesis	1.37E-02	4
Phenylpropanoids || Phytohormones	Salicylate biosynthesis	1.57E-03	3

*The genes were classified into three groups: **(A)** cultivars only (Ofir vs. Shiran); **(B)** mite treatment only (TSSM-infested vs. untreated control); and **(C)** overlap (both cultivar and TSSM-induced treatment). The enrichment analysis and pathway classification were performed using MetGenMAP (p < 0.05). Pathway name indicates the specific pathway, and supper class indicates the general metabolic classification according to SolCyc database (http://solcyc.solgenomics.net/)*.

As presented in Table 1C, only a few pathways within the overlap group were significantly enriched, including the initial reaction of phenylpropanoids toward flavonols and suberin biosynthesis, as well as the biosynthesis of the phytohormone, SA. Together, these observations revealed the contribution of cultivars to the variation, which was mainly associated with secondary metabolism. In contrast, the mite-inducible changes were related to both primary and secondary metabolism. The overlap group, which represents genes that differ by both cultivar type and infestation treatment, is related to secondary metabolism. GO enrichment for each of these groups showed similar results to Table 1, with a few additional categories. The GO enrichment summary of biological process, molecular function, and cellular component is presented in the Supplementary Table 4. There was at least one pathway in all the groups involved in the metabolism of volatile compounds, including monoterpenes, salicylate (and MeSA), and volatile benzenoid ester biosynthesis. Therefore, we further explored these two pathways, looking at gene expression and measuring volatile composition changes.

### Comparing the Expression of Genes Associated With the Terpene Pathway and Salicylic Acid Biosynthesis

The terpene biosynthesis gene list was generated according to a recently published study characterizing the tomato *Terpene synthase* (TPS) gene family by Zhou and Pichersky (2020) and *Apocarotenoid cleavage dioxygenase* (CCD) by Ilg et al. (2014). Out of 51 genes, 23 were detected in our dataset. As shown in Table 2A, five *Terpene synthase* (TPS) genes were highly expressed in untreated Ofir vs. Shiran, including *TPS5* (Solyc01g105890), *TPS7* (Solyc01g105920), and *TPS19/20* (Solyc08g005665) involved in monoterpene biosynthesis, *TPS9* (Solyc06g059885) involved in monoterpene and sesquiterpene biosynthesis, and *TPS18* (Solyc08g005720) involved in diterpene biosynthesis. This revealed the constitutive transcriptomic difference between the two tomato cultivars. Upon mite-infestation in the Ofir cultivar, the expression levels of six genes were reduced, including *TPS4* (Solyc01g105880), *TPS12* (Solyc06g059930), *Geranylgeranyl diphosphate synthases* (GGPPSs; Solyc04g079960 and Solyc02g085700), *Solanesyl diphosphate synthase* (SPPS; Solyc07g061990), and *Carotenoid cleavage dioxygenase* (CCD2; Solyc01g087260), while *TPS46* (Solyc03g006550) was significantly overexpressed. In Shiran cultivar, three genes were underexpressed (*TPS35*, Solyc01g101210; *GGPPS2*, Solyc04g079960 and *CCD2*, Solyc01g087260), and two genes were overexpressed (*TPS5*, Solyc01g105890; and *TPS46*, Solyc03g006550).

**Table 2:** Changes in the expression of genes involved in the biosynthesis of terpenoid and salicylic acid pathways.

			**Ofir-con/Shiran-con**		**Ofir-inf/Shiran-inf**		**Ofir-inf/Ofir-con**		**Shiran-inf/Shiran-con**	
**Class**	**Short name**	**Gene ID**	**log2 (FC)**	***p*****-value (FDR)**	**log2 (FC)**	***p*****-value (FDR)**	**log2 (FC)**	***p*****-value (FDR)**	**log2 (FC)**	***p*****-value (FDR)**
**(A)**										
Monoterpenes	TPS4	Solyc01g105880	1.278	0.372	-0.943	0.263	−1.603	**0.019**	0.618	0.432
	TPS5	Solyc01g105890	4.087	**0.005**	2.0348	0.991	0.388	0.647	2.44	**<0.001**
	TPS7	Solyc01g105920	6.016	**0.032**	5.504	0.104	−1.336	0.327	-0.825	0.678
	TPS19/20	Solyc08g005665	5.683	**0.005**	0.473	0.750	−1.954	0.146	3.256	0.2
	TPS25	Solyc02g079890	0.58	0.558	1.6005	0.474	−0.079	0.79	-1.1	0.478
Monoterpenes/ sesquiterpenes	TPS9	Solyc06g059885	1.632	**0.049**	1.1901	0.463	−0.681	0.247	-0.239	0.639
	TPS12	Solyc06g059930	1.498	0.308	0.414	0.860	−1.968	**0.037**	-0.883	0.432
Sesquiterpenes	TPS16	Solyc07g008690	0.24	0.577	0.595	0.686	−1.728	0.143	-2.083	0.719
	TPS17	Solyc12g006570	-0.18	0.572	0.338	0.991	−0.555	0.38	-1.074	0.799
	TPS27	Solyc00g154480	2.671	0.535	0.796	0.996	−0.454	0.999	1.421	0.192
	TPS35	Solyc01g101210	0.513	0.584	1.0367	0.151	−0.673	0.676	-1.197	**0.009**
Diterpenes	TPS18	Solyc08g005720	1.706	**0.032**	1.3328	0.463	−0.807	0.247	-0.435	0.432
	TPS21	Solyc08g005640	1.779	0.372	1.8287	0.903	−2.908	0.095	-2.958	0.432
	TPS24	Solyc07g066670	-0.403	0.52	0.390	0.463	0.802	**0.025**	0.009	0.901
	TPS24	Solyc07g066675	-0.387	0.663	-0.174	0.699	0.543	0.19	0.329	0.456
	TPS40	Solyc06g084240	0.656	0.765	-0.414	0.991	0.556	0.911	1.626	0.678
	TPS46	Solyc03g006550	0.728	0.535	0.555	0.534	5.004	**0.006**	5.177	**0.003**
Mono/diterpenes	GGPPS2	Solyc04g079960	0.566	0.372	0.290	0.910	−2.384	**0.013**	-2.108	**0.009**
	GGPPS3	Solyc02g085700	0.155	0.663	-0.242	0.463	−1.22	**0.007**	-0.823	0.228
	SSU II	Solyc09g008920	0.123	0.694	-0.334	0.151	−0.791	**0.039**	-0.334	0.363
Diterpenes	SPPS	Solyc07g061990	-0.55	0.558	-0.123	0.903	−1.083	**0.037**	-1.51	0.072
Tetraterpene cleavage	CCD1	Solyc01g087250	-0.897	0.53	0.069	0.686	0.181	0.083	-0.785	0.432
	CCD2	Solyc01g087260	0.071	0.734	-0.085	0.951	−1.61	**0.007**	-1.454	**0.003**
**(B)**										
SA biosynthesis (from chorismate)	ICS	Solyc06g071030	0.735	0.535	0.751	0.283	−1.504	**0.003**	-1.52	0.432
SA biosynthesis II (from Phe)	PAL	Solyc10g011920	1.689	0.372	1.7907	0.104	2.064	0.065	1.963	**0.014**
	PAL	Solyc03g036470	4.854	0.372	6.2477	0.120	5.879	0.05	4.486	**0.019**
	PAL	Solyc03g042560	1.742	0.535	7.0157	0.151	7.369	**0.039**	2.095	0.363
	PAL	Solyc05g056170	-0.363	0.558	0.502	0.463	1.455	**0.024**	0.59	0.306
	PAL	Solyc09g007900	0.366	0.558	-0.069	0.991	3.333	**0.004**	3.768	**0.013**
	PAL	Solyc09g007910	0.511	0.558	-0.103	0.991	1.049	**0.041**	1.663	0.148
	PAL	Solyc10g086180	-0.115	0.663	-0.275	0.991	−0.406	0.697	-0.246	0.481
Volatile benzenoid ester biosynthesis	BCLA	Solyc12g044300	0.582	0.138	-0.082	0.945	−1.663	**0.007**	-0.999	**0.013**
	BEBT	Solyc05g015800	1.551	0.115	-0.271	0.995	−6.432	**0.008**	-4.61	**0.013**
	BEBT	Solyc07g049660	0.571	0.53	-0.061	0.991	1.984	**0.003**	2.616	**0.016**
	BEBT	Solyc07g049670	0.476	0.558	0.181	0.903	2.127	**0.007**	2.423	**0.04**
	BEBT	Solyc08g005760	-0.268	0.558	0.164	0.991	−0.747	0.446	-1.178	**0.013**
	BEBT	Solyc11g020640	1.418	0.372	0.117	0.991	−1.93	0.083	-0.63	0.723
	CNL	Solyc02g081360	-0.037	0.97	-0.427	0.686	3.089	**0.007**	3.479	**0.013**
	CNL	Solyc03g031870	2.678	0.558	1.4372	0.134	2.172	0.143	3.413	**0.03**
	SAMT	Solyc01g081340	-0.08	0.663	0.312	0.699	−2.018	**0.019**	-2.41	0.218
	SAMT	Solyc02g084950	-1.105	0.372	-0.657	0.344	0.409	0.065	-0.039	0.857
	SAMT	Solyc04g055260	0.005	0.988	0.047	0.991	0.078	0.697	0.036	0.678
	SAMT	Solyc09g091550	-0.748	0.68	0.454	0.991	4.245	**0.037**	3.042	0.06

***(A)** Genes of the terpene biosynthesis were selected according to Ilg et al. (2014) and Zhou and Pichersky (2020). TPS, terpene synthase; GGPPS, geranylgeranyl diphosphate synthase; SSU II, small subunit of geranyl diphosphate synthase; FPPS, farnesyl diphosphate synthase; SPPS, solanesyl diphosphate synthase; and CCD, carotenoid cleavage dioxygenase. **(B)** Genes of salicylic acid (SA) and volatile benzenoid ester pathways were generated from LycoCyc and MetaCyc databases. ICS, isochorismate synthase; PAL, Phe ammonia lyase; BCLA, benzoate-CoA ligase; BEBT, benzoyl coenzyme A: benzyl alcohol benzoyl transferase; CNL, cinnamate:CoA ligase, and SAMT, salicylate 1-O-methyltransferase. Color coding indicates log2(FC) of induction (red) or repression (blue) values of trimmed mean of M-values (TMM) of average n = 3–4 (log2 >|1|, p < 0.05 FDR). Highlighted in bold are significantly different p-values*.

Notably, the gene ID of *TPS20* and *TPS19* was Solyc08g005665.1, Solyc08g005670.2, and then Solyc08g005665.1 again in *S. lycopersicum* annotation versions 2.4, 3.2, and 4.0, respectively. Both gene IDs have been used in the literature (Xu et al., 2018; Zhou and Pichersky, 2020). The current annotation suggests that *TPS19* and *TPS20* are two coding sequences on the same transcript and are likely splice variants. However, their high sequence similarity prevented the distinction between the two during the mapping phase. Therefore, to separate them in the current RNAseq data, their amino acid variation from the reference genome (Heinz. cv) was used to infer their presence in the raw sequencing reads. A few reads that showed amino acid profiles congruent with both proteins, were excluded. The quantification of the reads revealed that both isoforms are present in Ofir and Shiran (Supplementary Figure 4). In all four groups, *TPS20* had more reads than *TPS19*. Unlike the TMM analysis, where *TPS19/20* was not detected in control samples of Shiran cultivar (Supplementary Table 5), a few reads were detected in these samples.

Salicylic acid (SA) is synthesized via two pathways: (i) chorismate through *Isochorismate synthase* (ICS) and (ii) from Phe via *Phe ammonia-lyase* (PAL) (Lefevere et al., 2020). The gene list was generated from LycoCyc (http://solcyc.solgenomics.net/) and MetaCyc databases (Caspi et al., 2018). The results of the genes involved in SA and volatile benzenoid ester biosynthesis are shown in Table 2A. In Ofir, the expression levels of 11 genes from the Phe pathway were altered upon mite infestation, including *PALs* (Solyc03g042560, Solyc05g056170, Solyc09g007900, and Solyc09g007910), *Benzoate-CoA ligase* (BCLA; Solyc12g044300), *Benzyl alcohol benzoyl transferase* (BEBT; Solyc05g015800, Solyc07g049660, and Solyc07g049670), *Cinnamate:CoA ligase* (CNL; Solyc02g081360), and *Salicylate 1-O-methyltransferase* (SAMT; Solyc01g081340 and Solyc09g091550), and the expression of *ICS* (Solyc06g071030) from the chorismate pathway was significantly decreased. In Shiran, the expression levels of 9 genes from the Phe pathway were altered, including *PALs* (Solyc10g011920, Solyc03g036470, and Solyc09g007900), *BEBTs* (Solyc05g015800, Solyc07g049660, Solyc07g049670, and Solyc08g005760), and *CNLs* (Solyc02g081360 and Solyc03g031870), while the level of *ICS* did not change. While several genes were significantly modified in response to mite-infestation, none of these genes were significantly different between the two cultivars' untreated samples (constitutive differences), suggesting that the SA pathway is mostly involved in the inducible defense mechanisms. Altogether, the results indicate a dramatic change in the gene expression levels of SA and volatile benzenoid ester biosynthesis, mostly affected by mite treatment (inducible manner). Additionally, only a slight effect on the terpene biosynthesis pathway was seen, which was enriched more in the Ofir than the Shiran cultivar (constitutive manner).

### Content of Volatile Organic Compounds (VOCs)

The transcriptome analysis suggested that one of the major differences between these cultivars was related to the metabolism of VOCs. The two cultivars' volatile content was determined under both mite-infested and untreated control conditions using the HS-SPME-GC-MS technique to link the transcriptomic to the metabolic changes. A total of 54 metabolites were identified across all different treatments, including 12 monoterpenes, 7 sesquiterpenes, 8 irregular terpenes, 7 phenylpropanoids and benzenoids, and 20 aliphatics (Supplementary Table 6). In a principal component analysis, the majority of variance in volatiles content was due to the cultivar factor (PC1, 68.7% of total variation). The TSSM infestation factor (PC2) explained another 10% of the total variation in the data, reflecting the induced change in the volatiles content (Supplementary Figure 5).

To compare the relative levels of VOCs, Student's *t*-tests were conducted. The values in log2 fold change, separated into five volatile biosynthetic classes, are presented in Table 3. This analysis revealed that nine compounds from the mono-, and irregular terpenes differed between the two cultivars in both conditions (mite-infested and untreated control), including 4-carene, limonene, p-cymene, p-cymenene, terpinolene, α-terpinene, β-phellandrene, β-pinene, and crypton. However, only two compounds, α-pinene and o-guaiacol, were modified in the untreated Ofir vs. untreated Shian, and three compounds, 1-nonanol, 1-Octanol, and methyl salicylate, were altered in mite-infested Ofir vs. mite-infested Shiran. Methyl benzoate was the only compound that increased upon mite infestation in Shiran cultivar, while none of the metabolites changed upon mite infestation in the Ofir cultivar. Notably, two monoterpenes 3,7,7-trimethyl-1,3,5-cycloheptatriene and β-myrcene were detected only in the Ofir cultivar. These comparisons suggested that monoterpenes are the main VOCs that differ between cultivars (constitutive levels), while only minor changes occur upon TSSM attack.

**Table 3:** Changes in the mean abundance of volatiles between TSSM-infested/untreated tomato cultivars Ofir vs. Shiran.

		**Ofir-con/Shiran-con**		**Ofir-inf/Shiran-inf**		**Ofir-inf/Ofir-con**		**Shiran-inf/Shiran-con**	
**Class**	**Compund Name**	**Log2 (FC)**	***p*****-value (FDR)**	**Log2 (FC)**	***p*-value (FDR)**	**Log2 (FC)**	***p*****-value (FDR)**	**Log2 (FC)**	***p*-value (FDR)**
Monoterpenes	3,7,7-Trimethyl-1,3,5-cycloheptatriene					0.83	0.270		
	4-Carene	4.79	** <0.001**	6.66	**<0.001**	0.63	0.270	-1.24	0.485
	Limonene	3.14	** <0.001**	3.92	** <0.001**	0.68	0.270	-0.10	0.899
	p-Cymene	3.16	** <0.001**	3.74	** <0.001**	0.67	0.448	0.09	0.899
	p-Cymenene	2.52	**0.003**	3.06	** <0.001**	0.38	0.631	-0.16	0.899
	Terpinolene	1.66	**0.042**	2.39	** <0.001**	0.70	0.270	-0.03	0.899
	α-Pinene	-1.16	**0.030**	-0.71	0.269	0.62	0.292	0.18	0.899
	α-Terpinene	3.80	**0.002**	4.81	** <0.001**	0.46	0.395	-0.54	0.852
	β-Myrcene					0.54	0.431		
	β-phellandrene	3.72	** <0.001**	4.93	** <0.001**	0.51	0.270	-0.70	0.559
	β-Pinene	-2.20	**0.004**	-1.86	**0.015**	0.49	0.448	0.15	0.899
	γ-Terpinene	0.14	0.584	0.75	0.086	0.70	0.071	0.09	0.899
Sesquiterpenes	Alloaromadendrene	1.07	0.059	1.00	0.223	0.38	0.825	0.46	0.282
	Guaiazulene	0.53	0.349	1.06	0.144	0.97	0.358	0.44	0.485
	α-Humulene	1.13	0.050	1.07	0.125	0.49	0.631	0.55	0.261
	β-Caryophyllen	0.90	0.063	1.01	0.295	0.54	0.742	0.43	0.261
	β-Elemene	1.17	0.056	1.17	0.129	0.70	0.474	0.70	0.283
	β-trans-Caryophyllene	0.84	0.063	0.94	0.134	0.46	0.631	0.36	0.331
	δ-Elemene	-0.57	0.421	-0.06	0.894	0.82	0.270	0.30	0.899
Irregular terpenes	TMTT	1.28	0.175	1.25	0.086	0.70	0.431	0.73	0.500
	Crypton	3.18	** <0.001**	3.65	** <0.001**	0.64	0.270	0.17	0.899
	Dihydroactinidiolide	0.06	0.976	-0.06	0.845	0.21	0.631	0.34	0.424
	Hexahydrofarnesyl acetone	0.25	0.784	0.81	0.400	0.82	0.478	0.27	0.870
	β-Cyclocitral	0.54	0.164	0.20	0.303	0.26	0.478	0.60	0.038
	β-Homocyclocitral	0.40	0.510	0.23	0.223	0.33	0.474	0.50	0.161
	β-ionone	0.54	0.110	0.39	0.120	0.43	0.341	0.58	0.038
	β-Ionone epoxide	0.78	0.110	0.58	0.125	0.61	0.341	0.80	0.038
Aliphatics	1-Nonanol	-0.92	0.107	-1.62	**0.011**	-0.17	0.825	0.53	0.202
	1-Octanol	-0.47	0.175	-1.05	**0.011**	-0.25	0.742	0.33	0.202
	2,4-Heptadienal, E,E-	-0.03	0.804	0.22	0.430	0.09	0.742	-0.15	0.712
	2,4-Hexadienal	0.22	0.344	0.21	0.730	0.15	0.825	0.17	0.709
	2-Heptenal, Z-	0.38	0.461	0.57	0.303	0.13	0.843	-0.07	0.902
	2-Hexenal, E-	-0.16	0.183	-0.02	0.894	0.06	0.843	-0.08	0.709
	9,12,15-Octadecatrienoic acid, methyl ester, Z,Z,Z-	-0.92	0.293	-0.21	0.853	0.32	0.754	-0.39	0.852
	9,12-Octadecadienoic acid, methyl ester Z,Z-	-0.39	0.609	-0.15	0.894	0.16	0.825	-0.07	0.899
	E,E-2,4-Decadienal	0.77	0.609	1.83	0.057	1.03	0.396	-0.03	0.899
	Hexanoic acid methyl ester	1.21	0.115	0.74	0.086	-0.22	0.848	0.25	0.572
	Methyl hexadecanoate	0.26	0.528	0.43	0.129	0.26	0.448	0.08	0.899
	Methyl myristate	0.49	0.293	0.45	0.304	0.44	0.396	0.48	0.709
	Methyl nonanoate	0.06	0.944	0.13	0.704	0.54	0.448	0.47	0.899
	Methyl octadecanoate	0.16	0.857	0.52	0.303	0.41	0.448	0.05	0.899
	Methyl octanoate	0.76	0.115	0.07	0.832	0.03	0.843	0.72	0.117
	Methyl palmitoleate	-0.17	0.972	-0.69	0.894	0.12	0.825	0.64	0.899
	Methyl pentadecanoate	0.58	0.228	0.78	0.129	0.62	0.270	0.41	0.709
	Nonanal	0.08	0.784	-0.28	0.295	-0.10	0.742	0.27	0.572
Phenylpropanoids and benzenoids	2-Phenylethanol	0.20	0.461	0.06	0.705	-0.40	0.208	-0.27	0.572
	Benzaldehyde	0.54	0.155	0.10	0.598	-0.19	0.742	0.25	0.383
	Benzaldehyde, 3-ethyl-	0.09	0.804	0.17	0.705	0.22	0.691	0.14	0.899
	Benzyl alcohol	-0.01	0.920	-0.03	0.998	-0.02	0.957	0.00	0.902
	Methyl benzoate	0.49	0.555	-0.16	0.853	2.14	0.099	2.79	**0.023**
	Methyl salicylate	1.29	0.110	1.85	**0.022**	1.19	0.270	0.63	0.259
	o-Guaiacol	-1.38	**0.016**	-0.87	0.057	0.17	0.825	-0.34	0.572

*The average from five replicates produced on day 3 dpi was calculated and log-transformed for Student's t-test (df = 4; log2 >|1|, p < 0.05 FDR). Color coding indicates log2(FC) of induction (red) or repression (blue), and gray are VOCs that were not detected. TMTT, 4,8,12-trimethyltrideca-1,3,7,11-tetraene. Highlighted in bold are significantly different p-values*.

To compare the amount of VOCs in each cultivar and condition, we measured all compounds produced on day 3 dpi, and the change in each VOC was tested by a one-way ANOVA. Overall, 29 out of 54 VOCs varied significantly (*df* = 4; Fisher's LSD; *p* ≤ 0.05), indicating unique volatile content between the cultivars and treatments (Supplementary Table 7A). The most pronounced differences between the cultivars were in the levels of monoterpenes, of which 3,7,7-trimethyl-1,3,5-cycloheptatriene and β-myrcene were only detected in Ofir, and seven other monoterpenes were over-represented in Ofir relative to Shiran. Additionally, the most pronounced difference in response to TSSM infestation was in the level of the benzenoid compound methyl benzoate, which increased in response to TSSM infestation in both cultivars. Overall, MeSA was the most abundant molecule, followed by β-phellandrene in Ofir and methyl benzoate in Shiran (Supplementary Table 7B). To compare the total amount of VOCs in each cultivar and condition, we measured all 54 compounds produced. The total VOC content extracted from the Ofir control plants was approximately twice that of the Shiran plants. Upon infestation, both Ofir and Shiran plants produced ~1.5 times more VOCs (Supplementary Table 7B).

**Figure 5:**
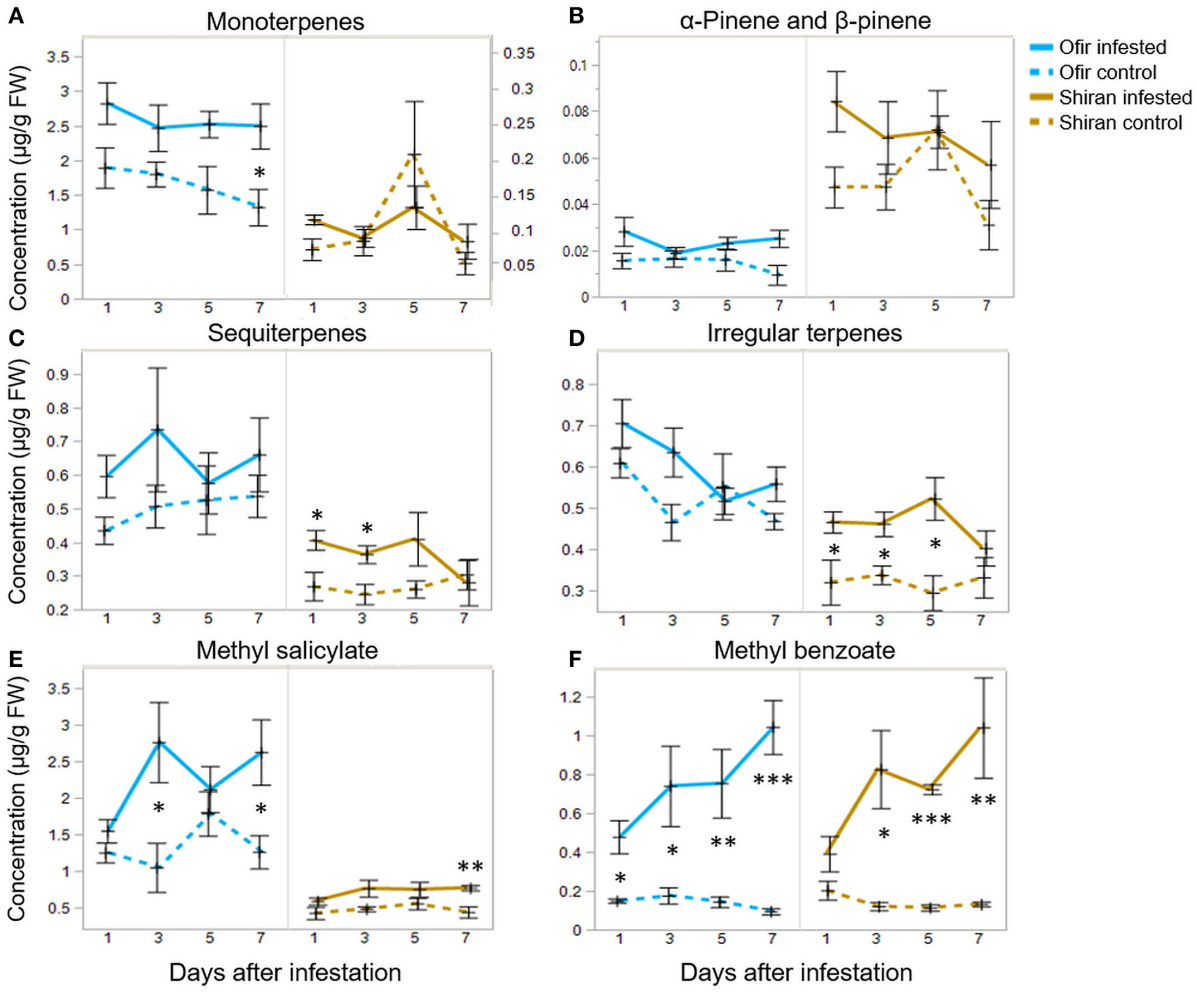
Volatile compound contents of tomato plants infested with TSSM or untreated (control) for 1, 3, 5, or 7 days. The graphs show the average relative production at each day for **(A)** monoterpenes, including the sum of 3,7,7-trimethyl-1,3,5-cycloheptatriene, 4-carene, α-terpinene, β-myrcene, β-phellandrene, p-cymene, p-cymenene, limonene, and terpinolene. **(B)** β-pinene, **(C)** sequiterpenes represent the sum of α-humulene, β-elemene, β-trans-caryophyllene, and guaiazulene. **(D)** Irregular terpenes represent the sum of β-cyclocitral, β-homocyclocitral β-ionone, β-ionone epoxide, and crypton. **(E)** Methyl salicylate (MeSA) and **(F)** methyl benzoate. Data represent mean ± SE; *n* = 4–5. Asterisks indicate significant differences between the control and the infested in each cultivar (Student's *t*-test, *p < 0.05, **p < 0.01, and ***p < 0.001).

In this dataset, Ofir and Shiran seemed to produce different volatile patterns, both in quality and quantity. The question was whether differences in these patterns were due to changes in specific classes of metabolites. To that end, the use of hierarchical cluster analysis (Ward's linkage) resulted in a heatmap, presented in Supplementary Figure 6. Overall, the VOC dataset was divided into six clusters, and volatiles mostly grouped by classes (monoterpenes, sesquiterpenes, and irregular terpenes). Six groups of metabolites were further selected, and their average levels were present in infested and untreated plants at different times (1, 3, 5, and 7 dpi) shown in Figure 5. The sum of 9 monoterpenes was more than 10 times higher in Ofir than in Shiran. Notably, the only monoterpenes in greater quantity in Shiran were α-pinene and β-pinene (Figures 5A,B, respectively). Both sesquiterpene and the irregular terpenes presented similar patterns between the groups and were higher in Ofir than in Shiran. However, irregular terpenes varied more between the Shiran control and infested treatments (Figures 5C,D, respectively). The volatile phytohormone MeSA was significantly higher in Ofir than in Shiran and increased in the infested treatments (Figure 5E). Methyl benzoate was the most prominent compounds that increased in response to TSSM infestation in both cultivars and was the only compound that increased over time (Figure 5F). VOCs associated with the aliphatic class did not show a clear pattern, and most of them were more abundant in Ofir than Shiran, except 1-nonanol, and 1-octanol, which presented the opposite trend (Table 3 and Supplementary Figure 6).

Further evidence for volatile differences between the two cultivars was discovered by measuring the VOC profile of tomatoes grown in the net house. Leaf samples were collected over 3 months, and volatiles were detected using GC-MS liquid extraction. The results suggested that Ofir accumulated higher amounts of volatiles during this time than in Shiran (Supplementary Figure 7). These findings supported our laboratory experiments, wherein the total amount of VOCs was higher in Ofir than Shiran (Supplementary Table 7B). Furthermore, 1 week after TSSM infestation, the amount of volatiles increased until 2 weeks after infestation. Subsequently, it declined during the establishment of the mite population (Figure 1 and Supplementary Figure 5, respectively). The VOC list is presented in Supplementary Table 8.

### Olfactometry Bioassay of TSSM and *Phytoseiulus persimilis* Upon Exposure to Shiran and Ofir Odors

**Figure 6:**
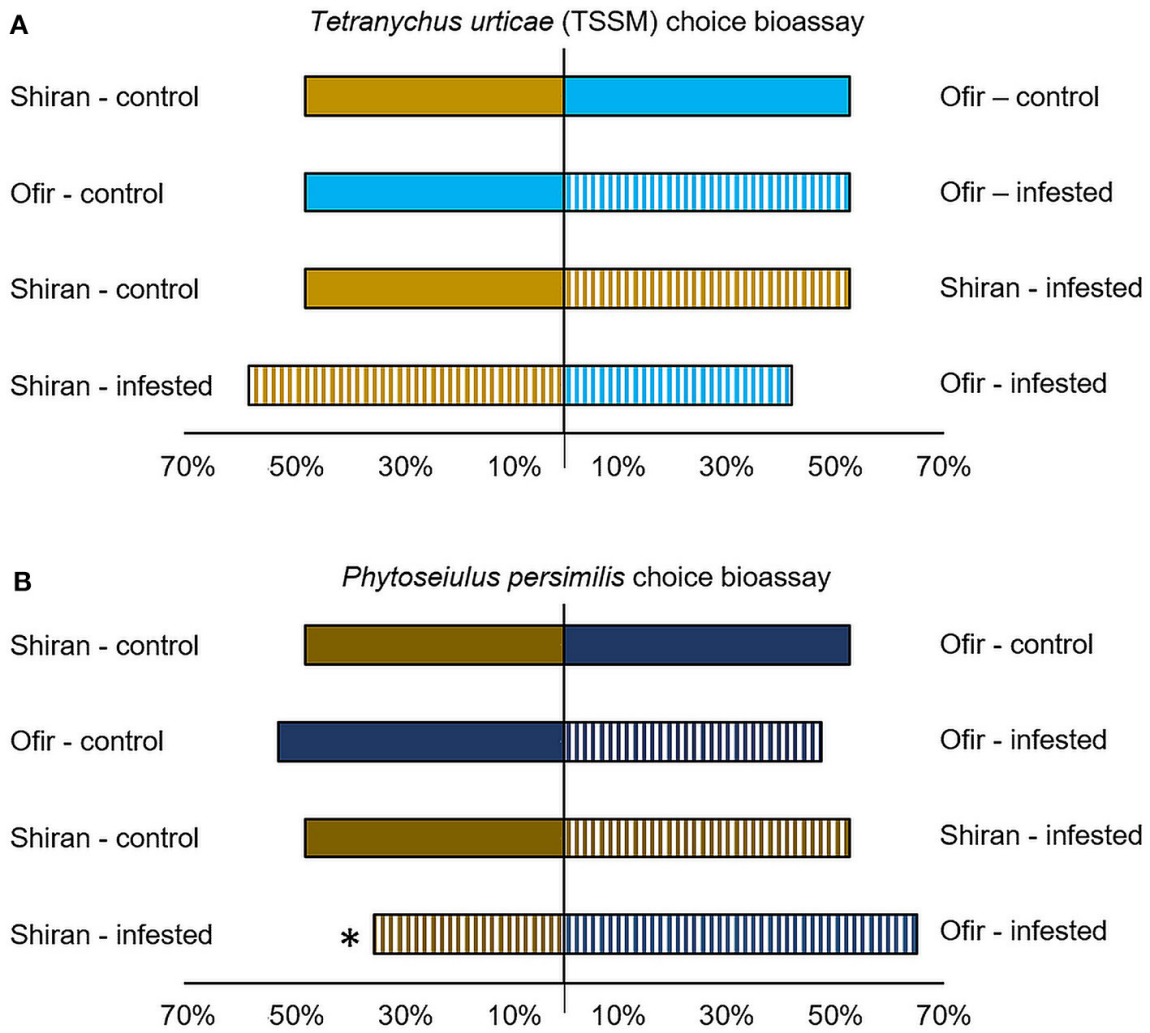
Olfactory response of *Tetranychus urticae* (TSSM) and *Phytoseiulus persimilis* to different tomato cultivars infested with TSSMs and untreated (control). **(A)** Percentage of TSSMs that chose the odors of infested plants (striped bars) for control plants (solid bars) or Ofir cultivar (light blue bars) for Shiran cultivar (light beige bars). **(B)** Percentage of *P. persimilis* that chose the odors of infested plants (striped bars) for control plants (solid bars) or Ofir cultivar (blue bars) for Shiran cultivar (beige bars). Experiments were repeated twice. In each experiment, 40 individual adult mites were tested (χ^2^-test, *p < 0.05).

A Y-shape olfactometry bioassay was designed (Figure 6) to understand the effect of different VOC emissions on mite behavior. The TSSMs showed no preference for the odor source emitted by intact Shiran-control and Ofir-control plants (χ^2^ = 0.31; *p* = 0.577). Similarly, TSSMs had no preference when given a choice between Ofir-control and Ofir-infested plants (χ^2^ = 0.31; *p* = 0.577), Shiran-control and Shiran-infested plants (χ^2^ = 0.13; *p* = 0.715), and Shiran-infested and Ofir-infested plants (χ^2^ = 0.78; *p* = 0.376; Figure 6A). *Phytoseiulus persimilis* predatory mite is known to respond to unique VOC compositions (Takabayashi et al., 2000). Therefore, we tested the indirect effect of the VOCs blend of the two cultivars on these mites by exposing them to either TSSM-infested or untreated (control) Ofir and Shiran volatile emissions. The *P. persimilis* clearly chose Ofir-infested over Shiran-infested plants (χ^2^ = 4.94; *p* = 0.0263; Figure 6B). *Phytoseiulus persimilis* did not show a significant preference when given a choice between Shiran-control and Ofir-control plants (χ^2^ = 0.31; *p* = 0.577), Ofir-control and Ofir-infested plants (χ^2^ = 0.31; *p* = 0.577), and Shiran-control and Shiran-infested plants (χ^2^ = 0.13; *p* = 0.715). The results indicated that the predator mite could distinguish between infested Ofir and Shiran, but the TSSM could not.

## Discussion

Considerable progress has been made in tomato plant cultivation by characterizing its volatile emissions during herbivore attack and investigating their crucial role in attracting natural enemies (Kant et al., 2004; Mayo-Hernández et al., 2019). However, the genetic basis underlying the constitutive release of VOCs involved in direct and indirect defenses, and their effect on herbivores, are still poorly understood. In this work, we compared the changes in volatile content and related gene expression in two commercial tomato cultivars and their effects on mites to identify the resistance mechanism. We characterized the variation of the TSSM population in the field and climate-controlled conditions and compared the transcriptomic and volatile profiles. Many studies have compared genotypes to reintroduce beneficial traits such as VOC emissions from wild species into domesticated lines (Bleeker et al., 2009; Mayo-Hernández et al., 2019; Paudel et al., 2019). Here, we focused on two cluster cherry tomato cultivars with similar physical (similar trichome density; Supplementary Figure 8) and agronomic traits (fruit size, shape, color, and cluster architecture), but with contrasting TSSM susceptibility to isolate chemical traits involved in mite-resistance. Beyond exploiting the chemical diversity of wild tomato species, we propose that there is an untapped potential within domesticated tomato cultivars for isolating and exploiting resistance traits due to the great genetic variation between them, including variation in monoterpene volatiles.

### Salicylic Acid and Terpenoid Biosynthesis Are Involved in Mite Responses in Tomato Leaves

One of the first plant physiological responses to TSSM infestation is stomatal closure, leading to reduced photosynthetic rates, chlorophyll content, transpiration efficiency, and overall yield (Reddall et al., 2004). As a result of mite infestation, primary metabolism (i.e., amino acids, sugar and starch biosynthesis, photosynthesis, and chloroplast-related metabolism) is modified, in addition to changes in secondary metabolites and phytohormones (i.e., SA, JA, and ET; Martel et al., 2015). Via transcriptome analysis, we revealed that the expression of specific genes was affected by TSSM infestation in the leaves of Shiran and Ofir tomato cultivars. Notably, only a few pathways were significantly enriched by the differences between cultivars alone. These genes were associated with primary and secondary metabolism, such as terpenoid and phenylpropanoid pathways (Table 1). One class of over-represented terpenoids was monoterpenes, which reportedly varies between cultivars in a constitutive as well as inducible manner (Raghava et al., 2010). The second class of terpenoids enriched by TSSM infestation was tetraterpenoids (related to carotenoids and abscisic acid). Carotenoids serve as pigments and degrade into irregular volatile terpenoids (i.e., β-ionone), previously reported to be induced in response to TSSM infestation (Nyalala et al., 2013). Genes related to SA biosynthesis were significantly enriched in the group overlapping cultivars and mite infestation. SA and MeSA are generally known to accumulate upon infection with biotrophic pathogens (Wei et al., 2014) and infestation with herbivores such as phloem-sap-sucking insects (Zarate et al., 2007) and TSSM (Kawazu et al., 2012; Thaler et al., 2012). Notably, in our analysis, the JA pathway was not enriched based on the over-representation and GO enrichment analysis (Table 1 and Supplementary Table 4). Previous reports indicated that the crosstalk between SA-JA was important for VOC biosynthesis and emission. However, the relationship between the two phytohormones still requires investigation. A reciprocal antagonistic relationship between SA and JA has been described in at least 17 different plant species (Thaler et al., 2012), and their levels were manipulated by both herbivores and pathogens (Martel et al., 2015). On the other hand, a few other reports have shown that both SA and JA biosynthetic and signaling genes were enhanced upon TSSM infestation in tomatoes (Kawazu et al., 2012). This suggested that the SA-JA crosstalk is species-specific, cultivar-specific, and can vary between different responses (Kappers et al., 2010; Thaler et al., 2012; Wei et al., 2014; Rioja et al., 2017).

### TSSM Induces SA-Related Gene Expression, Whereas Constitutive Changes Are Associated With Terpenoid Biosynthesis

Plants synthesize SA via two pathways, *Isochorismate synthase* (ICS) and *Phe ammonia-lyase* (PAL), both originating from chorismate. However, not all enzymes catalyzing SA have been identified. The importance of these two pathways varies in different plant species (Lefevere et al., 2020). In *Arabidopsis*, for instance, the *ICS* pathway is more active; in rice, the *PAL* pathway seems to be more dominant, while in soybean, both pathways contribute equally to SA accumulation (Silverman et al., 1995; Duan et al., 2014). In our study, transcriptional changes in TSSM-induced genes were associated with *PAL* degradation (via phenylpropanoids), whereas *ICS* (Solyc06g071030) expression was only reduced upon TSSM infestation in the Ofir cultivar. Similar results were previously reported in tomato (cv. Heinz 1706) and pepper (Martel et al., 2015; Zhang et al., 2020). Terpene synthases genes *TPS5, TPS7, TPS9, TPS18*, and *TPS19/20*, were expressed more in the TSSM-resistant cultivar (Ofir) vs. Shiran. Our findings indicated that SA-pathway-associated gene expression was induced upon infestation, whereas terpenoid biosynthetic genes varied more between cultivars and were more constitutively higher in Ofir than it Shiran.

### Quantity and Diversity of Volatiles Play an Important Role in Determining Resistance to TSSMs

The transcriptome analysis revealed that several pathways involved in VOC biosynthesis were modified in response to mite infestation, including SA, benzenoid esters, and terpenoid biosynthesis (Table 2). These results were consistent with the change in volatile compounds. Numerous metabolites originating from these classes were previously shown to confer repellent or toxic properties. Methyl benzoate (benzenoid class), for instance, exerts significant contact toxicity against TSSM eggs and adult females and displays repellent activity against adult mites (Mostafiz et al., 2020). In our study, methyl benzoate was increased upon infestation in both cultivars.

**Figure 7:**
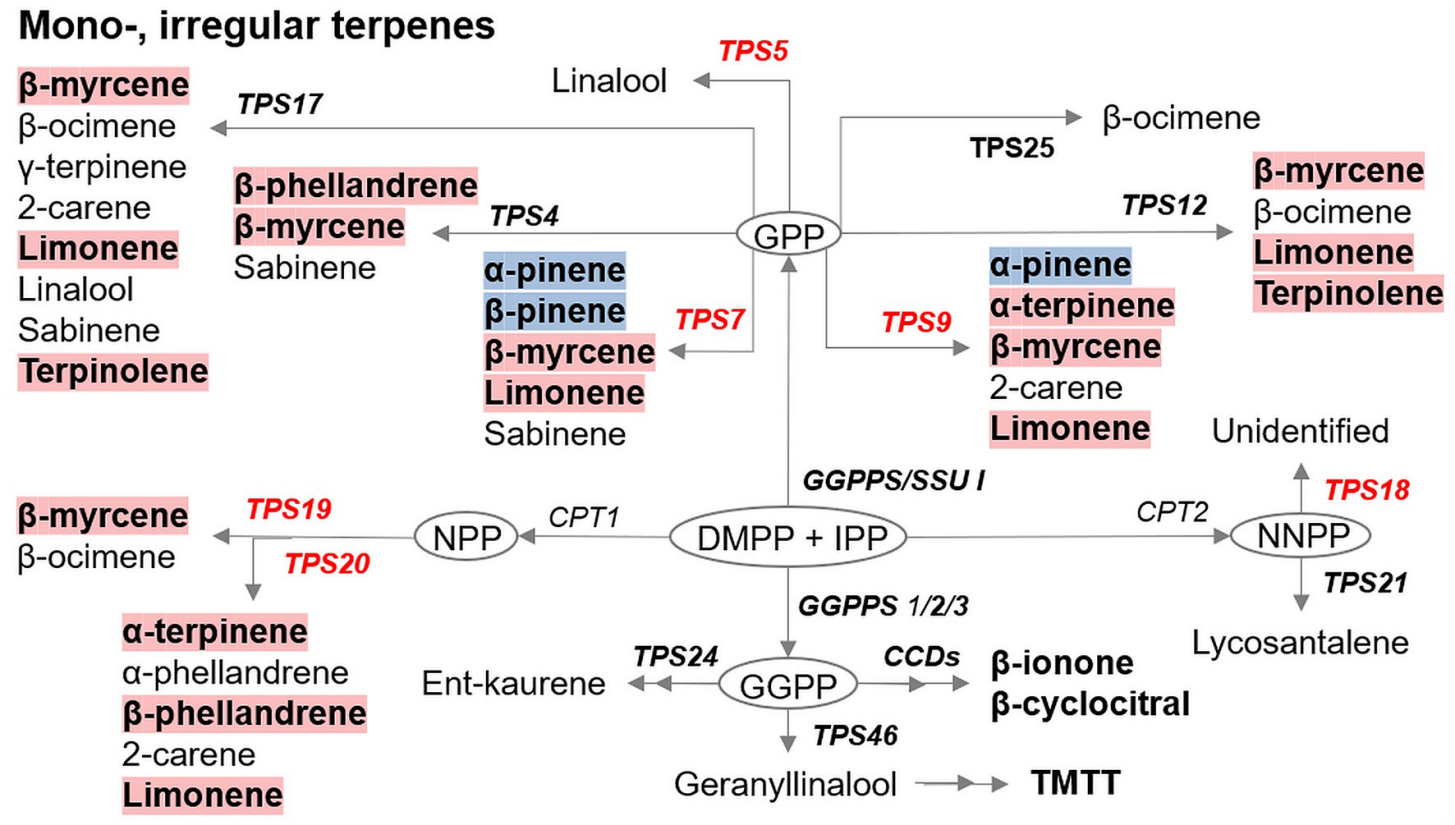
Summary of the significant differences in gene expression of the terpene biosynthesis in the leaves of two tomato cultivars. Genes are shown in italics, red-colored expressed higher in the resistant cultivar (Ofir) than in the susceptible cultivar (Shiran). Volatiles with red background produced more in Ofir, and volatiles with blue background produced more in Shiran. Gene and volatiles identified in this work are shown in bold. CPT, cis-prenyltransferase; DMAPP, dimethylallyl diphosphate; E,E-FPP, trans-farnesyl diphosphate; FPPS, E,E-FPP synthase; GPP, geranyl diphosphate; GGPP, geranylgeranyl diphosphate; GGPPS, GGPP synthase; IPP, isopentenyl diphosphate; NPP, neryl diphosphate; NNPP, nerylneryl diphosphate; SSU, small subunit of GGPPS; TPS, terpene synthase; Z,Z-FPP, cis-farnesyl diphosphate.

The majority of VOCs detected in our experiments were terpenoids. Figure 7 presents a summary of changes in genes and metabolites associated with mono-, and irregular terpenoid pathway in uninfested Ofir vs. Shiran cultivars as presented in Tables 2, 3. From a total of 54 VOCs, 11 monoterpenes, one irregular terpene and five *Terpene synthases* genes were modified in the resistant cultivar (Ofir) relative to the susceptible cultivar (Shiran). Although many genes involved in the production of volatiles and their regulation in tomato are known (Zhou and Pichersky, 2020), many details remain unresolved. Some of the terpenoids identified in our results were not shown in the summary scheme due to a lack of previous research. The previous report had indicated that the release of TSSM herbivore-induced plant volatiles (HIPV) from sour orange (*Citrus aurantium*) triggered defense responses in another citrus plant, *Cleopatra mandarin*. This HIPV was dominantly composed of terpenes α-ocimene, α-farnesene, pinene, and limonene, and the green leaf volatile 4-hydroxy-4-methyl-2-pentanone (Agut et al., 2015). Limonene (monoterpene) was described in determining mite reproduction of TSSMs and was reported to possess acaricidal activity (Roh et al., 2013; Abdelgaleil et al., 2019). Other monoterpenes, *p*-cymene, α-terpinene, and β-phellandrene, were identified as repellent compounds in whitefly bioassays (Bleeker et al., 2009) while *p*-cymene was also reported to repel western flower thrips (Janmaat et al., 2002). Exogenous application of two irregular terpenes, β-cyclocitral and β-ionone, reduced TSSM activity (Nyalala et al., 2013). These data are consistent with our results: all terpenoids mentioned above (limonene, *p*-cymene, α-terpinene, β-phellandrene, β-cyclocitral, and β-ionone) were higher in the resistant cultivar than in the susceptible one (Figure 7). *TPS7* and *TPS19/20* genes were significantly different between the two cultivars and were expressed more in the resistant cultivar (Ofir). *TPS7* encodes enzymes known to catalyze the formation of several monoterpenes, namely, β-myrcene, β-pinene, and limonene. β-myrcene and limonene were higher in Ofir as expected by the higher expression of *TPS7*; however, α-pinene and β-pinene levels were lower in Ofir compared to Shiran (Figure 7). These findings may suggest differential preferences of *TPS7* toward some substrates rather than others by a yet unknown gene mechanism. Previous studies highlighted the importance of *TPS20* (catalyzing the formation of several monoterpenes, namely, α-terpinene, β-phellandrene, and limonene) and its alleles in terpene biosynthesis and chemical diversity in the glandular trichomes of tomato, and its wild relative *Solanum habrochaites* (Schilmiller et al., 2009; Gonzales-Vigil et al., 2012). These reports combined with our findings suggest that in our selected tomato cultivars, *TPS7* and *TPS19/20* genes are important for determining mite resistance. Additionally, *TPS5* was detected in Ofir, but the catabolic product, linalool, was not detected.

### The Different Effect of VOCs on Phytoseiulus Persimilis and Tetranychus Urticae

Variation in volatile blends is known to play a crucial role in processes such as natural enemy attraction (Kant et al., 2004). The predator mite, *P. persimilis*, is known to rely on olfactory cues to locate its prey (Van Den Boom et al., 2004; Kappers et al., 2011). However, the effect of olfactory cues on TSSMs is less studied. A previous study of citrus rootstocks demonstrated a TSSM preference using a similar olfactometer system (Agut et al., 2015), which helped to guide our decision to test the TSSM olfactory choice. TSSMs showed no preference in cultivar choice, suggesting that volatile blends are less effective in long-distance repellency. Our results from the *P. persimilis* olfactory choice assays showed that the predator mite was more attracted to the volatile blend of TSSM-infested Ofir than TSSM-infested Shiran. Nevertheless, *P. persimilis* showed no preference between either untreated cultivar or between infested and untreated Ofir. It is impossible to determine which of the volatile components affects *P. persimilis* attraction without isolating each compound. Previous studies have confirmed that MeSA is a key *P. persimilis* attractant (De Boer and Dicke, 2004; De Boer et al., 2008). Our data shows that MeSA was markedly higher in the resistant cultivar and was significantly induced after 3 days of TSSM infestation (the day that the Y-tube bioassay was conducted) as presented in Supplementary Table 7. MeSA was significantly different between the infested Shiran and Ofir plants. These differences might explain the *P. persimilis* preference in our results.

Altogether, the expression levels of genes involved in the terpenoid biosynthesis and differences in terpenoid volatile contents of tomato cultivars with different mite resistance levels, suggest that this chemical class plays an important role in the resistance of tomatoes to TSSM infestation. Further investigation is required to understand the source of these differences, for example, transcription factor expression and regulation and genetic variation, among other factors.

The TSSM olfactory choice assays showed that TSSMs are not repelled by the volatile blend. However, the colony size, TSSM performance, and fecundity experiments reinforce the notion that Ofir is more resistant than Shiran. If we couple the VOC and RNA-seq datasets with the literature showing direct effects of volatiles such as limonene, α-terpinene, and β-phellandrene on oviposition or feeding, it is quite likely that volatile blends may at least partially contribute to close-contact-related mite resistance in tomatoes. To validate this, we will further need to manipulate specific VOC levels via gene knock-down of over-expression, together with targeted metabolomics to reveal non-volatile compounds that potentially confer Ofir resistance (e.g., acyl-sugars and alkaloids).

## Conclusion

Pests are responsible for substantial crop losses worldwide by destroying plant tissues and depleting their resources (direct damage), as well as transmitting plant diseases (indirect damage) (Douglas, 2018). Multiple strategies such as plant breeding and genetic modifications are being used to improve crop resistance to pests, including optimization of defense mechanisms. This process requires a knowledge of specific defense mechanisms and their regulation. Our results may serve as the basis for breeding and developing new management strategies for the TSSM, based on plant volatile emissions to improve defense mechanisms. One strategy that may be used is developing markers for TSSM resistance. However, further molecular and fieldwork is needed to increase our understanding of the precise resistance mechanism, focusing on the constitutive levels and defense induction that confers this resistance between the cultivars.

## Data Availability Statement

The datasets presented in this study can be found in online repositories. The names of the repository/repositories and accession number(s) can be found in the article/Supplementary Material.

## Author Contributions

NW, AC, AS, LA, IO, and VT conceived and designed the experiments. NW, AC, AS, LA, and IO performed the experiments. NW, AC, BY, and VT analyzed the data and contributed to the writing of the manuscript. All authors contributed to the article and approved the submitted version.

## Conflict of Interest

The authors declare that the research was conducted in the absence of any commercial or financial relationships that could be construed as a potential conflict of interest.

## References

[B1] Abdelgaleil S. A. M. Badawy M. E. I. Mahmoud N. F. Marei A. E. S. M. (2019). Acaricidal activity, biochemical effects and molecular docking of some monoterpenes against two-spotted spider mite (*Tetranychus urticae* Koch). Pestic. Biochem. Physiol. 156, 105–115. 10.1016/j.pestbp.2019.02.006 31027569

[B2] Agut B. Gamir J. Jacas J. A. Hurtado M. Flors V. (2014). Different metabolic and genetic responses in citrus may explain relative susceptibility to *Tetranychus urticae*. Pest Manag. Sci. 70, 1728–1741. 10.1002/ps.3718 24375985

[B3] Agut B. Gamir J. Jaques J. A. Flors V. (2015). *Tetranychus urticae*-triggered responses promote genotype-dependent conspecific repellence or attractiveness in citrus. New Phytol. 207, 790–804. 10.1111/nph.13357 25771705

[B4] Aharoni A. Jongsma M. A. Bouwmeester H. J. (2006). Volatile science? Metabolic engineering of terpenoids in plants. Trends Plant Sci. 10, 18–24. 10.1016/j.tplants.2005.10.005 16290212

[B5] Alexa A. Rahnenführer J. Lengauer T. (2006). Improved scoring of functional groups from gene expression data by decorrelating GO graph structure. Bioinformatics 22, 1600–1607. 10.1093/bioinformatics/btl140 16606683

[B6] Ament K. Kant M. R. Sabelis M. W. Haring M. A. Schuurink R. C. (2004). Jasmonic acid is a key regulator of spider mite-induced volatile terpenoid and methyl salicylate emission in tomato. Plant Physiol. 135, 2025–2037. 10.1104/pp.104.048694 15310835 PMC520773

[B7] Ament K. Van Schie C. C. Bouwmeester H. J. Haring M. A. Schuurink R. C. (2006). Induction of a leaf specific geranylgeranyl pyrophosphate synthase and emission of (E,E)-4,8,12-trimethyltrideca-1,3,7,11-tetraene in tomato are dependent on both jasmonic acid and salicylic acid signaling pathways. Planta 224, 1197–1208. 10.1007/s00425-006-0301-5 16786318

[B8] Ameye M. Allmann S. Verwaeren J. Smagghe G. Haesaert G. Schuurink R. C.. (2018). Green leaf volatile production by plants: a meta-analysis. New Phytol. 220, 666–683. 10.1111/nph.14671 28665020

[B9] Bai Y. Lindhout P. (2007). Domestication and breeding of tomatoes: what have we gained and what can we gain in the future? Ann. Bot. 100, 1085–1094. 10.1093/aob/mcm150 17717024 PMC2759208

[B10] Bensoussan N. Santamaria M. E. Zhurov V. Diaz I. Grbić M. Grbić V. (2016). Plant-herbivore interaction: dissection of the cellular pattern of *Tetranychus urticae* feeding on the host plant. Front. Plant Sci. 7:1105. 10.3389/fpls.2016.01105 27512397 PMC4961969

[B11] Bleeker P. M. Diergaarde P. J. Ament K. Guerra J. Weidner M. Schütz S.. (2009). The role of specific tomato volatiles in tomato-whitefly interaction. Plant Physiol. 151, 925–935. 10.1104/pp.109.142661 19692533 PMC2754627

[B12] Bolland H. R. Gutierrez J. Flechtmann C. H. (1998). World Catalogue of the Spider Mite Family (Acari: Tetranychidae). Lieden: Brill Academic Publishers.

[B13] Cáceres L. A. Lakshminarayan S. Yeung K. K.-C. McGarvey B. D. Hannoufa A. Sumarah M. W.. (2016). Repellent and attractive effects of α-, β-, and dihydro-β- ionone to generalist and specialist herbivores. J. Chem. Ecol. 42, 107–117. 10.1007/s10886-016-0669-z 26852133

[B14] Caspi R. Billington R. Fulcher C. A. Keseler I. M. Kothari A. Krummenacker M.. (2018). The MetaCyc database of metabolic pathways and enzymes. Nucleic Acids Res. 46, D633–D639. 10.1093/nar/gkx935 29059334 PMC5753197

[B15] Cheynier V. Comte G. Davies K. M. Lattanzio V. Martens S. (2013). Plant phenolics: Recent advances on their biosynthesis, genetics, and ecophysiology. Plant Physiol. Biochem. 72, 1–20. 10.1016/j.plaphy.2013.05.009 23774057

[B16] De Boer J. G. Dicke M. (2004). Experience with methyl salicylate affects behavioural responses of a predatory mite to blends of herbivore-induced plant volatiles. Entomol. Exp. Appl. 110, 181–189. 10.1111/j.0013-8703.2004.00133.x

[B17] De Boer J. G. Hordijk C. A. Posthumus M. A. Dicke M. (2008). Prey and non-prey arthropods sharing a host plant: effects on induced volatile emission and predator attraction. J. Chem. Ecol. 34, 281–290. 10.1007/s10886-007-9405-z 18185960 PMC2266969

[B18] De Moraes C. M. Mescher M. C. Tumlinson J. H. (2001). Caterpillar-induced nocturnal plant volatiles repel conspecific females. Nature 410, 577–579. 10.1038/35069058 11279494

[B19] Douglas A. E. (2018). Strategies for enhanced crop resistance to insect pests. Annu. Rev. Plant Biol. 69, 637–660. 10.1146/annurev-arplant-042817-040248 29144774

[B20] Duan L. Liu H. Li X. Xiao J. Wang S. (2014). Multiple phytohormones and phytoalexins are involved in disease resistance to Magnaporthe oryzae invaded from roots in rice. Physiol. Plant. 152, 486–500. 10.1111/ppl.12192 24684436

[B21] Escobar-Bravo R. Alba J. M. Pons C. Granell A. Kant M. R. Moriones E.. (2016). A jasmonate-inducible defense trait transferred from wild into cultivated tomato establishes increased whitefly resistance and reduced viral disease incidence. Front. Plant Sci. 7:1732. 10.3389/fpls.2016.01732 27920785 PMC5118631

[B22] Escudero L. A. Ferragut F. (2005). Life-history of predatory mites *Neoseiulus californicus* and *Phytoseiulus persimilis* (Acari: Phytoseiidae) on four spider mite species as prey, with special reference to Tetranychus evansi (Acari: Tetranychidae). Biol. Control 32, 378–384. 10.1016/j.biocontrol.2004.12.010

[B23] Fernández-Muñoz R. Domínguez E. Cuartero J. (2000). A novel source of resistance to the two-spotted spider mite in Lycopersicon pimpinellifolium (Jusl.) Mill.: its genetics as affected by interplot interference. Euphytica 111, 169–173. 10.1023/A:1003893432676

[B24] Frost C. J. Mescher M. C. Carlson J. E. De Moraes C. M. (2008). Plant defense priming against herbivores: getting ready for a different battle. Plant Physiol. 146, 818–824. 10.1104/pp.107.113027 18316635 PMC2259053

[B25] Fürstenberg-Hägg J. Zagrobelny M. Bak S. (2013). Plant defense against insect herbivores. Int. J. Mol. Sci. 14, 10242–10297. 10.3390/ijms140510242 23681010 PMC3676838

[B26] Gonzales-Vigil E. Hufnagel D. E. Kim J. Last R. L. Barry C. S. (2012). Evolution of TPS20-related terpene synthases influences chemical diversity in the glandular trichomes of the wild tomato relative *Solanum habrochaites*. Plant J. 71, 921–935. 10.1111/j.1365-313X.2012.05040.x 22563774 PMC3466413

[B27] Gyan N. M. Yaakov B. Weinblum N. Singh A. Cna'ani A. Ben-Zeev S.. (2020). Variation between three Eragrostis tef accessions in defense responses to Rhopalosiphum padi aphid infestation. Front. Plant Sci. 11:1892. 10.3389/fpls.2020.598483 33363559 PMC7752923

[B28] He J. Bouwmeester H. J. Dicke M. Kappers I. F. (2020). Transcriptional and metabolite analysis reveal a shift in direct and indirect defences in response to spider-mite infestation in cucumber (*Cucumis sativus*). Plant Mol. Biol. 103, 507–509. 10.1007/s11103-020-01009-8 32306368 PMC7299927

[B29] Heil M. (2014). Herbivore-induced plant volatiles: targets, perception and unanswered questions. New Phytol. 204, 297–306. 10.1111/nph.12977

[B30] Ilg A. Bruno M. Beyer P. Al-Babili S. (2014). Tomato carotenoid cleavage dioxygenases 1A and 1B: Relaxed double bond specificity leads to a plenitude of dialdehydes, mono-apocarotenoids and isoprenoid volatiles. FEBS Open Bio. 4, 584–593. 10.1016/j.fob.2014.06.005 25057464 PMC4096678

[B31] Janmaat A. F. de Kogel W. J. Woltering E. J. (2002). Enhanced fumigant toxicity of p-cymene against *Frankliniella occidentalis* by simultaneous application of elevated levels of carbon dioxide. Pest Manag. Sci. 58, 167–173. 10.1002/ps.432 11852641

[B32] Kant M. R. Ament K. Sabelis M. W. Haring M. A. Schuurink R. C. (2004). Differential timing of spider mite-induced direct and indirect defenses in tomato plants. Plant Physiol. 135, 483–495. 10.1104/pp.103.038315 15122016 PMC429400

[B33] Kappers I. F. Hoogerbrugge H. Bouwmeester H. J. Dicke M. (2011). Variation in herbivory-induced volatiles among cucumber (*Cucumis sativus* L.) varieties has consequences for the attraction of carnivorous natural enemies. J. Chem. Ecol. 37, 150–160. 10.1007/s10886-011-9906-7 21249432 PMC3043237

[B34] Kappers I. F. Verstappen F. W. A. Luckerhoff L. L. P. Bouwmeester H. J. Dicke M. (2010). Genetic variation in jasmonic acid- and spider mite-induced plant volatile emission of cucumber accessions and attraction of the predator *Phytoseiulus persimilis*. J. Chem. Ecol. 36, 500–512. 10.1007/s10886-010-9782-6 20383796 PMC2866305

[B35] Kawazu K. Mochizuki A. Sato Y. Sugeno W. Murata M. Seo S.. (2012). Different expression profiles of jasmonic acid and salicylic acid inducible genes in the tomato plant against herbivores with various feeding modes. Arthropod. Plant. Interact. 6, 221–230. 10.1007/s11829-011-9174-z

[B36] Keeling C. I. Bohlmann J. (2006). Genes, enzymes and chemicals of terpenoid diversity in the constitutive and induced defence of conifers against insects and pathogens. New Phytol. 170, 657–675. 10.1111/j.1469-8137.2006.01716.x 16684230

[B37] Keskin N. Kumral N. A. (2015). Screening tomato varietal resistance against the two-spotted spider mite [*Tetranychus urticae* (Koch)]. Int. J. Acarol. 41, 300–309. 10.1080/01647954.2015.1028440

[B38] Kessler A. (2017). “Plant defences against herbivore attack,” in eLS (John Wiley & Sons, Ltd.). 10.1002/9780470015902.a0001324.pub3

[B39] Kessler A. Baldwin I. T. (2001). Defensive function of herbivore-induced plant volatile emissions in nature. Science 291, 2141–2144. 10.1126/science.291.5511.2141 11251117

[B40] Khalequzzaman M. Mondal M. Haque M. Karim M. (1970). Predatory efficacy of *Phytoseiulus persimilis* Athias-Henriot (Acari: Phytoseiidae) on the two spotted spider mite *Tetranychus urticae* Koch (Acari: Tetranychidae). J. Bio-Sci. 15, 127–132. 10.3329/jbs.v15i0.2152

[B41] Kumari P. Cna'ani A. Didi-Cohen S. Tzin V. Khozin-Goldberg I. (2020). Nitrogen deprivation-induced production of volatile organic compounds in the arachidonic-acid-accumulating microalga lobosphaera incisa underpins their role as ROS scavengers and chemical messengers. Front. Mar. Sci. 7, 1–21. 10.3389/fmars.2020.0041032802822

[B42] Lefevere H. Bauters L. Gheysen G. (2020). Salicylic acid biosynthesis in plants. Front. Plant Sci. 11:338. 10.3389/fpls.2020.00338 32362901 PMC7182001

[B43] Love M. I. Huber W. Anders S. (2014). Moderated estimation of fold change and dispersion for RNA-seq data with DESeq2. Genome Biol. 15:550. 10.1186/s13059-014-0550-8 25516281 PMC4302049

[B44] Markus Lange B. Ahkami A. (2013). Metabolic engineering of plant monoterpenes, sesquiterpenes and diterpenes-current status and future opportunities. Plant Biotechnol. J. 11, 169–196. 10.1111/pbi.12022 23171352

[B45] Martel C. Zhurov V. Navarro M. Martinez M. Cazaux M. Auger P.. (2015). Tomato whole genome transcriptional response to *Tetranychus urticae* Identifies divergence of spider mite-induced responses between tomato and arabidopsis. Mol. Plant-Microbe Interact. 28, 343–361. 10.1094/MPMI-09-14-0291-FI 25679539

[B46] Mayo-Hernández J. Ramírez-Chávez E. Molina-Torres J. Guillén-Cisneros M. L. Rodríguez-Herrera R. Hernández-Castillo F.. (2019). Effects of bactericera cockerelli herbivory on volatile emissions of three varieties of *Solanum lycopersicum*. Plants 8:509. 10.3390/plants8110509 31731734 PMC6918368

[B47] Migeon A. Ferragut F. Escudero-Colomar L. A. Fiaboe K. Knapp M. De Moraes G. J.. (2009). Modelling the potential distribution of the invasive tomato red spider mite, Tetranychus evansi (Acari: Tetranychidae). Exp. Appl. Acarol. 48, 199–212. 10.1007/s10493-008-9229-8 19153813

[B48] Mostafiz M. M. Shim J. K. Hwang H. S. Bunch H. Lee K. Y. (2020). Acaricidal effects of methyl benzoate against *Tetranychus urticae* Koch (Acari: Tetranychidae) on common crop plants. Pest Manag. Sci. 10.1002/ps.5770 32003105

[B49] Nyalala S. O. Petersen M. A. Grout B. W. W. (2013). Volatile compounds from leaves of the African spider plant (Gynandropsis gynandra) with bioactivity against spider mite (*Tetranychus urticae*). Ann. Appl. Biol. 162, 290–298. 10.1111/aab.12021

[B50] Pallini A. Janssen A. Sabelis M. W. (1997). Odour-mediated responses of phytophagous mites to conspecific and heterospecific competitors. Oecologia 110, 179–185. 10.1007/s004420050147 28307422

[B51] Park Y.-L. Lee J.-H. (2002). Leaf cell and tissue damage of cucumber caused by two spotted spider mite (Acari: Tetranychidae). J. Econ. Entomol. 95, 952–957. 10.1093/jee/95.5.952 12403421

[B52] Paudel S. Lin P.-A. Foolad M. R. Ali J. G. Rajotte E. G. Felton G. W. (2019). Induced plant defenses against herbivory in cultivated and wild tomato. J. Chem. Ecol. 45, 693–707. 10.1007/s10886-019-01090-4 31367970

[B53] Pérez-Hedo M. Arias-Sanguino Á. M. Urbaneja A. (2018). Induced tomato plant resistance against *Tetranychus urticae* triggered by the phytophagy of nesidiocoris tenuis. Front. Plant Sci. 9, 1–8. 10.3389/fpls.2018.01419 30333844 PMC6175976

[B54] Raghava T. Ravikumar P. Hegde R. Kush A. (2010). Spatial and temporal volatile organic compound response of select tomato cultivars to herbivory and mechanical injury. Plant Sci. 179, 520–526. 10.1016/j.plantsci.2010.07.020 21802610

[B55] Rakha M. Bouba N. Ramasamy S. Regnard J.-L. Hanson P. (2017). Evaluation of wild tomato accessions (Solanum spp.) for resistance to two-spotted spider mite (*Tetranychus urticae* Koch) based on trichome type and acylsugar content. Genet. Resour. Crop Evol. 64, 1011–1022. 10.1007/s10722-016-0421-0

[B56] Reddall A. Sadras V. O. Wilson L. J. Gregg P. C. (2004). Physiological responses of cotton to two-spotted spider mite damage. Crop Sci. 44, 835–846. 10.2135/cropsci2004.8350

[B57] Rioja C. Zhurov V. Bruinsma K. Grbic M. Grbic V. (2017). Plant-herbivore interactions: a case of an extreme generalist, the two-spotted spider mite *Tetranychus urticae*. Mol. Plant-Microbe Interact. 30, 935–945. 10.1094/MPMI-07-17-0168-CR 28857675

[B58] Robinson M. D. Oshlack A. (2010). A scaling normalization method for differential expression analysis of RNA-seq data. Genome Biol. 11:R25. 10.1186/gb-2010-11-3-r25 20196867 PMC2864565

[B59] Roh H. S. Lee B. H. Park C. G. (2013). Acaricidal and repellent effects of myrtacean essential oils and their major constituents against *Tetranychus urticae* (Tetranychidae). J. Asia. Pac. Entomol. 16, 245–249. 10.1016/j.aspen.2013.03.001

[B60] Santamaria M. E. Arnaiz A. Rosa-Diaz I. González-Melendi P. Romero-Hernandez G. Ojeda-Martinez D. A.. (2020). Plant defenses against *Tetranychus urticae*: mind the gaps. Plants 9:464. 10.3390/plants9040464 32272602 PMC7238223

[B61] Santamaria M. E. Martínez M. Cambra I. Grbic V. Diaz I. (2013). Understanding plant defence responses against herbivore attacks: an essential first step towards the development of sustainable resistance against pests. Transgenic Res. 22, 697–708. 10.1007/s11248-013-9725-4 23793555

[B62] Schauer N. Zamir D. Fernie A. R. (2005). Metabolic profiling of leaves and fruit of wild species tomato: a survey of the *Solanum lycopersicum* complex. J. Exp. Bot. 56, 297–307. 10.1093/jxb/eri057 15596477

[B63] Schilmiller A. L. Schauvinhold I. Larson M. Xu R. Charbonneau A. L. Schmidt A.. (2009). Monoterpenes in the glandular trichomes of tomato are synthesized from a neryl diphosphate precursor rather than geranyl diphosphate. Proc. Natl. Acad. Sci. U.S.A. 106, 10865–10870. 10.1073/pnas.0904113106 19487664 PMC2705607

[B64] Silverman P. Seskar M. Kanter D. Schweizer P. Metraux J. P. Raskin I. (1995). Salicylic acid in rice (biosynthesis, conjugation, and possible role). Plant Physiol. 108, 633–639. 10.1104/pp.108.2.633 12228500 PMC157383

[B65] Takabayashi J. Shimoda T. Dicke M. Ashihara W. Takafuji A. (2000). Induced response of tomato plants to injury by green and red strains of *Tetranychus urticae*. Exp. Appl. Acarol. 24, 377–383. 10.1023/A:1006497024175 11156163

[B66] Thaler J. S. Humphrey P. T. Whiteman N. K. (2012). Evolution of jasmonate and salicylate signal crosstalk. Trends Plant Sci. 17, 260–270. 10.1016/j.tplants.2012.02.010 22498450

[B67] Tzin V. Galili G. (2010). New Insights into the shikimate and aromatic amino acids biosynthesis pathways in plants. Mol. Plant 3, 956–972. 10.1093/mp/ssq048 20817774

[B68] Van Den Boom C. E. M. Van Beek T. A. Posthumus M. A. De Groot A. Dicke M. (2004). Qualitative and quantitative variation among volatile profiles induced by *Tetranychus urticae* feeding on plants from various families. J. Chem. Ecol. 30, 69–89. 10.1023/B:JOEC.0000013183.72915.99 15074658

[B69] Van Leeuwen T. Vontas J. Tsagkarakou A. Dermauw W. Tirry L. (2010). Acaricide resistance mechanisms in the two-spotted spider mite *Tetranychus urticae* and other important Acari: a review. Insect Biochem. Mol. Biol. 40, 563–572. 10.1016/j.ibmb.2010.05.008 20685616

[B70] War A. R. Paulraj M. G. Ahmad T. Buhroo A. A. Hussain B. Ignacimuthu S.. (2012). Mechanisms of plant defense against insect herbivores. Plant Signal. Behav. 7, 1306–1320. 10.4161/psb.21663 22895106 PMC3493419

[B71] Wei J. van Loon J. J. A. Gols R. Menzel T. R. Li N. Kang L.. (2014). Reciprocal crosstalk between jasmonate and salicylate defence-signalling pathways modulates plant volatile emission and herbivore host-selection behaviour. J. Exp. Bot. 65, 3289–3298. 10.1093/jxb/eru181 24759882 PMC4071845

[B72] Weintraub P. Palevsky E. (2008). Evaluation of the predatory mite, *Neoseiulus californicus*, for spider mite control on greenhouse sweet pepper under hot arid field conditions. Exp. Appl. Acarol. 45, 29–37. 10.1007/s10493-008-9169-3 18584132

[B73] Xia J. Psychogios N. Young N. Wishart D. S. (2009). MetaboAnalyst: a web server for metabolomic data analysis and interpretation. Nucleic Acids Res. 37, 652–660. 10.1093/nar/gkp356 19429898 PMC2703878

[B74] Xu J. van Herwijnen Z. O. Dräger D. B. Sui C. Haring M. A. Schuurink R. C. (2018). SlMYC1 regulates type VI glandular trichome formation and terpene biosynthesis in tomato glandular cells. Plant Cell 30, 2988–3005. 10.1105/tpc.18.00571 30518626 PMC6354261

[B75] Zarate S. I. Kempema L. A. Walling L. L. (2007). Silverleaf whitefly induces salicylic acid defenses and suppresses effectual jasmonic acid defenses. Plant Physiol. 143, 866–875. 10.1104/pp.106.090035 17189328 PMC1803729

[B76] Zhang Y. Bouwmeester H. J. Kappers I. F. (2020). Combined transcriptome and metabolome analysis identifies defence responses in spider mite-infested pepper (*Capsicum annuum*). J. Exp. Bot. 71, 330–343. 10.1093/jxb/erz422 31557301 PMC6913709

[B77] Zhou F. Pichersky E. (2020). The complete functional characterisation of the terpene synthase family in tomato. New Phytol. 226:1341–1360. 10.1111/nph.16431 31943222 PMC7422722

[B78] Zhurov V. Navarro M. Bruinsma K. A. Arbona V. Santamaria M. E. Cazaux M.. (2014). Reciprocal responses in the interaction between arabidopsis and the cell-content-feeding chelicerate herbivore spider mite. Plant Physiol. 164, 384–399. 10.1104/pp.113.231555 24285850 PMC3875816

