# Supplementary material for: Tomato Cultivars Resistant or Susceptible to Spider Mites Differ in Their Biosynthesis and Metabolic Profile of the Monoterpenoid Pathway
Source: Front Plant Sci. 2021 Feb 26;12:630155. doi: 10.3389/fpls.2021.630155 (PMC7952643; doi:10.3389/fpls.2021.630155)
Supplement: Supplementary file 1 [file Data_Sheet_1.PDF]

Figure S1

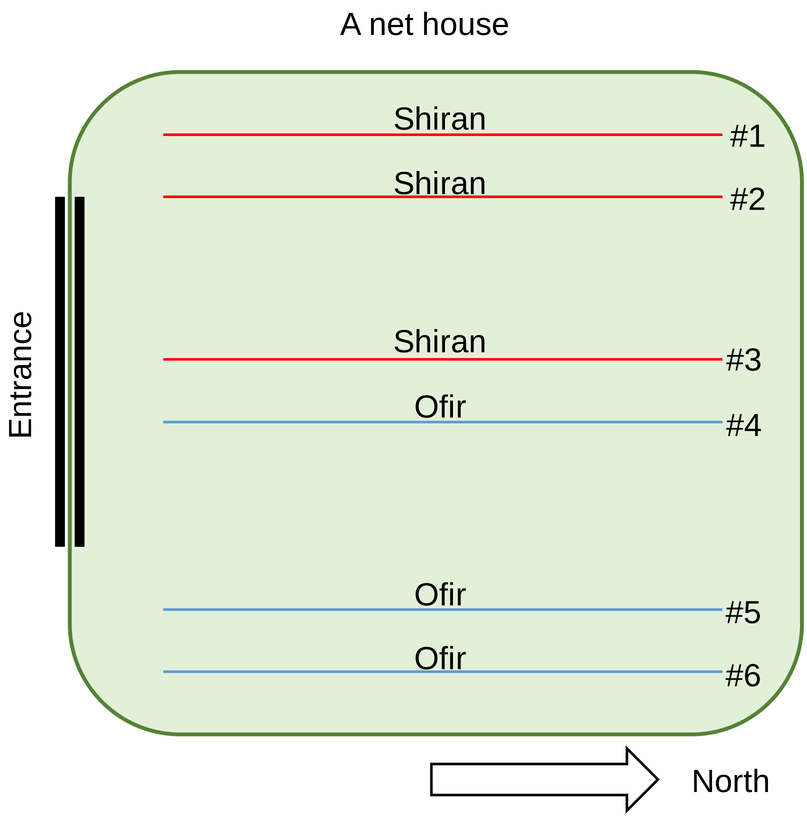

Figure S1. Illustration of the net house experimental design. Seedlings were transplanted and grown for three months with an irrigation system and treated with pesticides except for acaricides. From the second week after transplanting, the mite population was monitored. In each net house, three rows from each cultivar were planted. Each row includes 15 plants. Plants from rows 2 and 5 were examined for TSSM and plant damage, while rows 1, 3, 4 and 6 were planted to keep the same density has farmers use in this region.

Figure S2

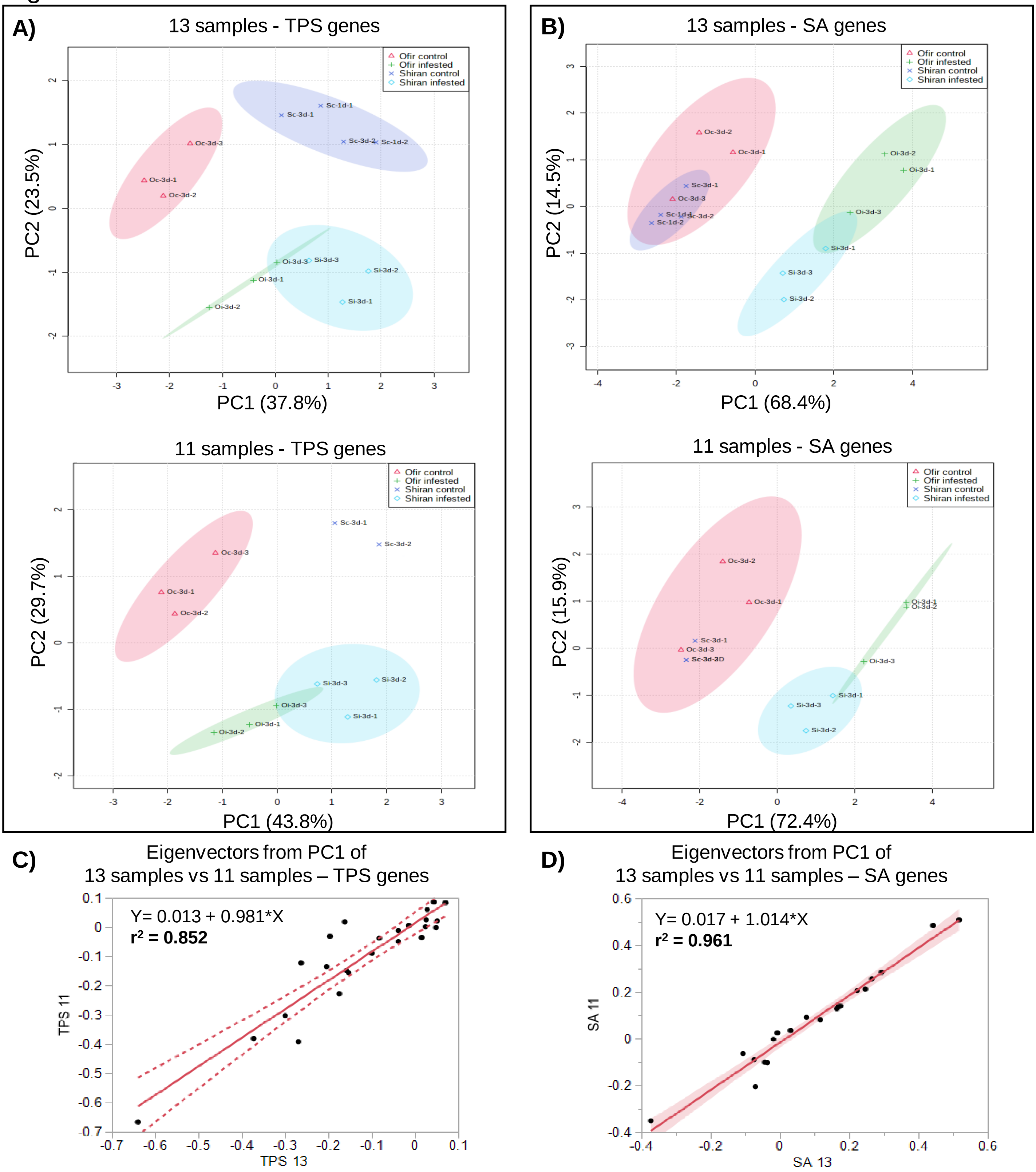

Figure S2. Principal component analysis (PCA) data of terpenoids and salicylic acid pathways genes. Five-weeks old tomato plants were either infested TSSM for 3 days (Oi-3d and Si-3d) or remained untreated as a control (Oc-3d, and Sc-3d). PCA plots of the TMM data generated for 13 samples, including three replicates of Ofir control (Oc-3d), Ofir infested (Oi-3d), Shiran infested (Si-3d). Shiran control samples (Sc) were sample either at 3d (Sc-3d, two samples) and/or at 1d (Sc-1d, two samples). PCA plots, including four Sc samples (a total of 13 samples) and only two Sc samples (a total of 11 samples). A) PCA of the 24 terpenoid biosynthetic genes, and B) PCA of the 20 salicylic acid biosynthetic genes. Correlation analysis of Eigenvectors from PC1 of C) terpenoids, or D) salicylic acid genes generated from 13 samples or 11 samples. Data were normalized by log2 transformation for the projection of PCA.

Figure S3

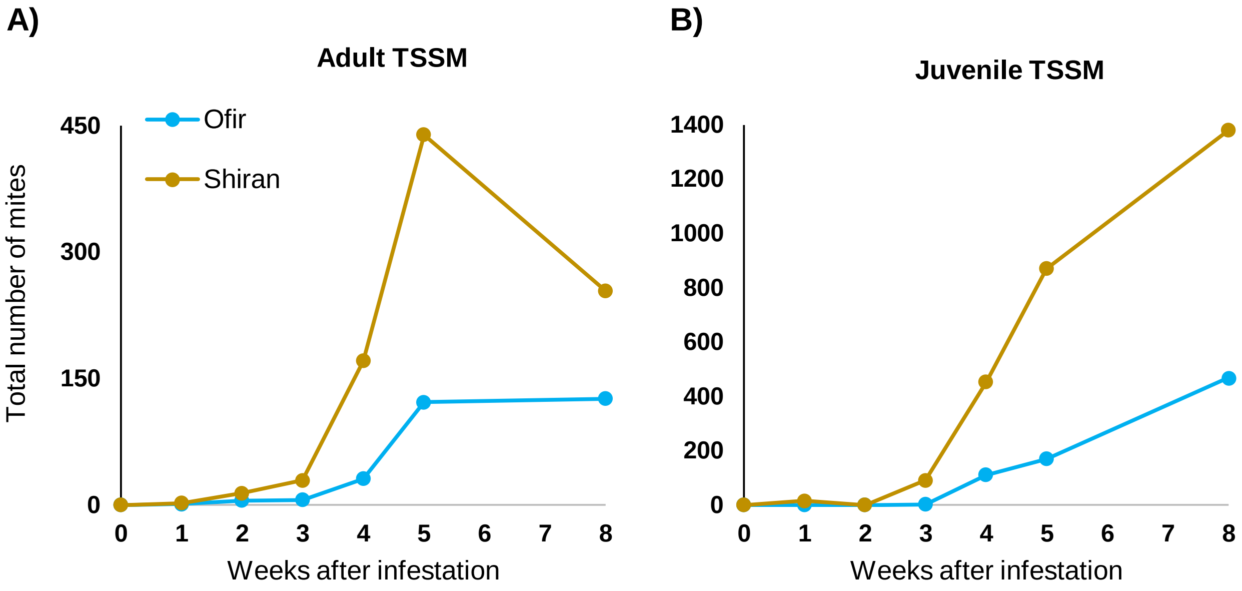

Figure S3. The total number of adult and juvenile TSSM on the two tomato cultivars. Mite population was counted from one till eight weeks after infestation. At each time point, nine leaves from a plant were selected, twenty plants of each cultivar were sampled. The total mite number at each time point is presented.

Figure S4

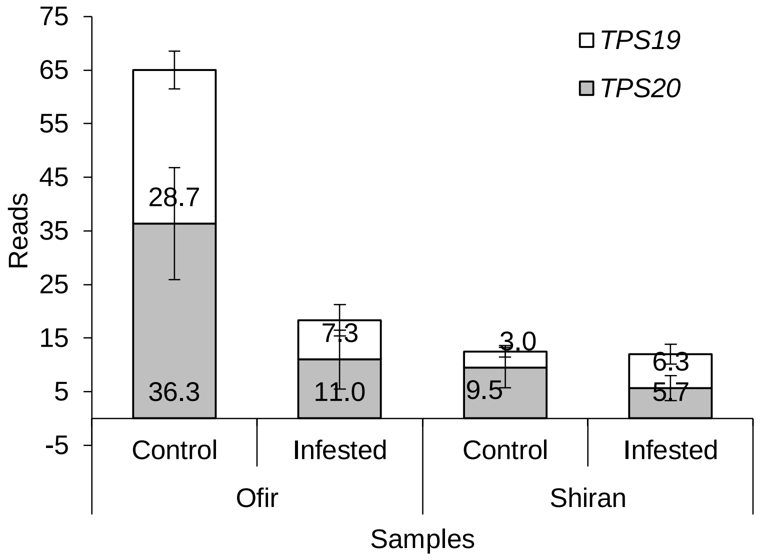

Figure S4. The average number of reads corresponding to *TPS19* or *TPS20.* Reads were detected using amino acid variations (see Materials and Methods) for RNAseq samples of Ofir and Shiran, under control and infested conditions. Mean +/- SE (n = 3-4).

Figure S5

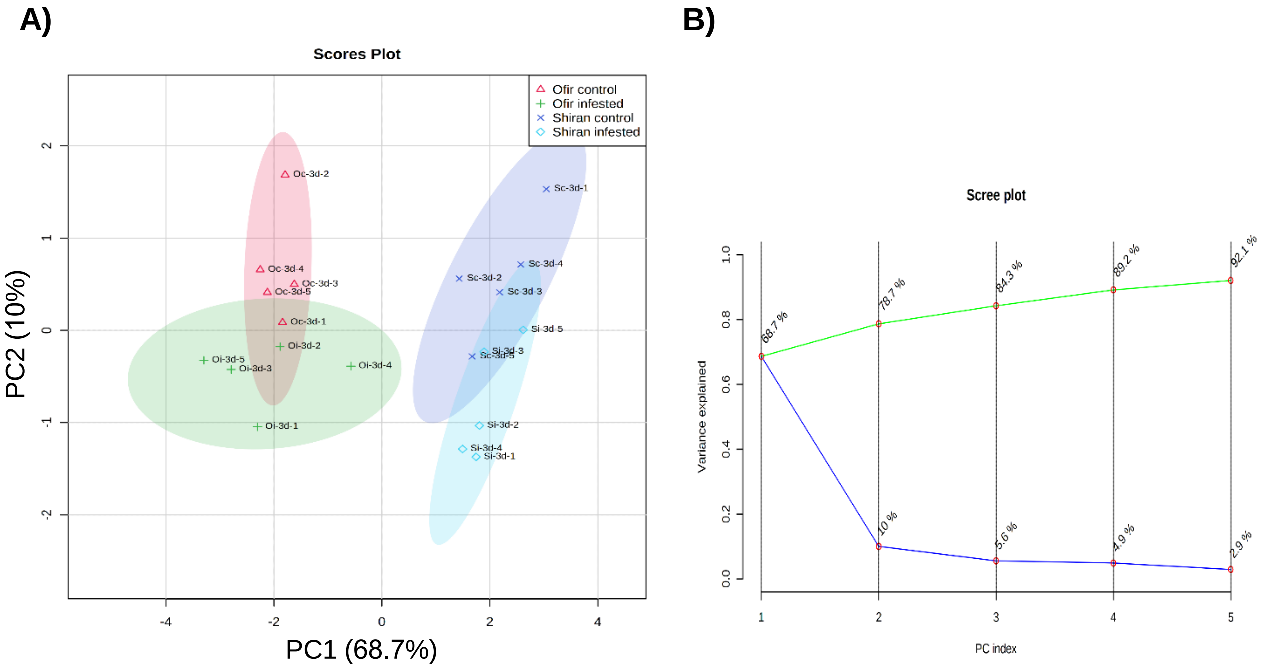

Figure S5. Principal component analysis (PCA) data of volatile compounds detected in the two different tomato cultivars, infested or untreated with TSSM for 3 days. (A) PCA plot component 1 and component 2. B) Scree plot shows the variance explained by PCs. The green line on top shows the accumulated variance explained; the blue line underneath shows the variance explained by individual PC.

Figure S6

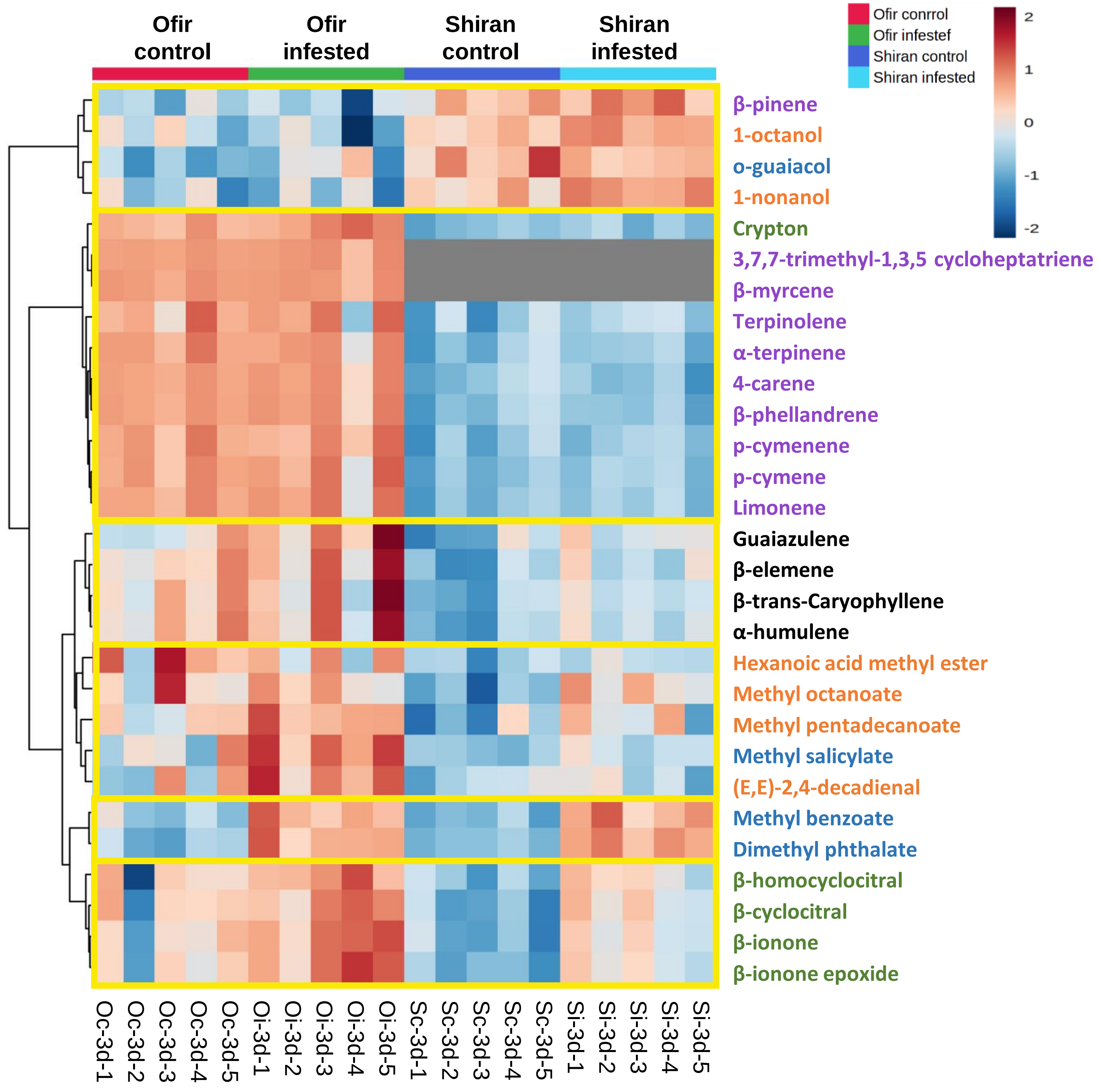

Figure S6. Hierarchical clustering analysis of volatile profiles of different tomato cultivars infested and control plants at day 3. The clustering result is shown as a heatmap from five replicates after log transformed (distance measure using euclidean, and clustering algorithm using Ward's linkage). Grey color indicates undetectable. Monoterpenes are shown in purple, irregular terpenes are shown in green, sesquiterpenes are shown in black, benzenoids are shown in blue and aldehydes are shown in orange. Yellow rectangles indicate the different clusters. Significant differences among treatments were calculated per compound with one-way ANOVA (df = 4; p ≤ 0.05). Only compounds with significant differences are presented.

Figure S7

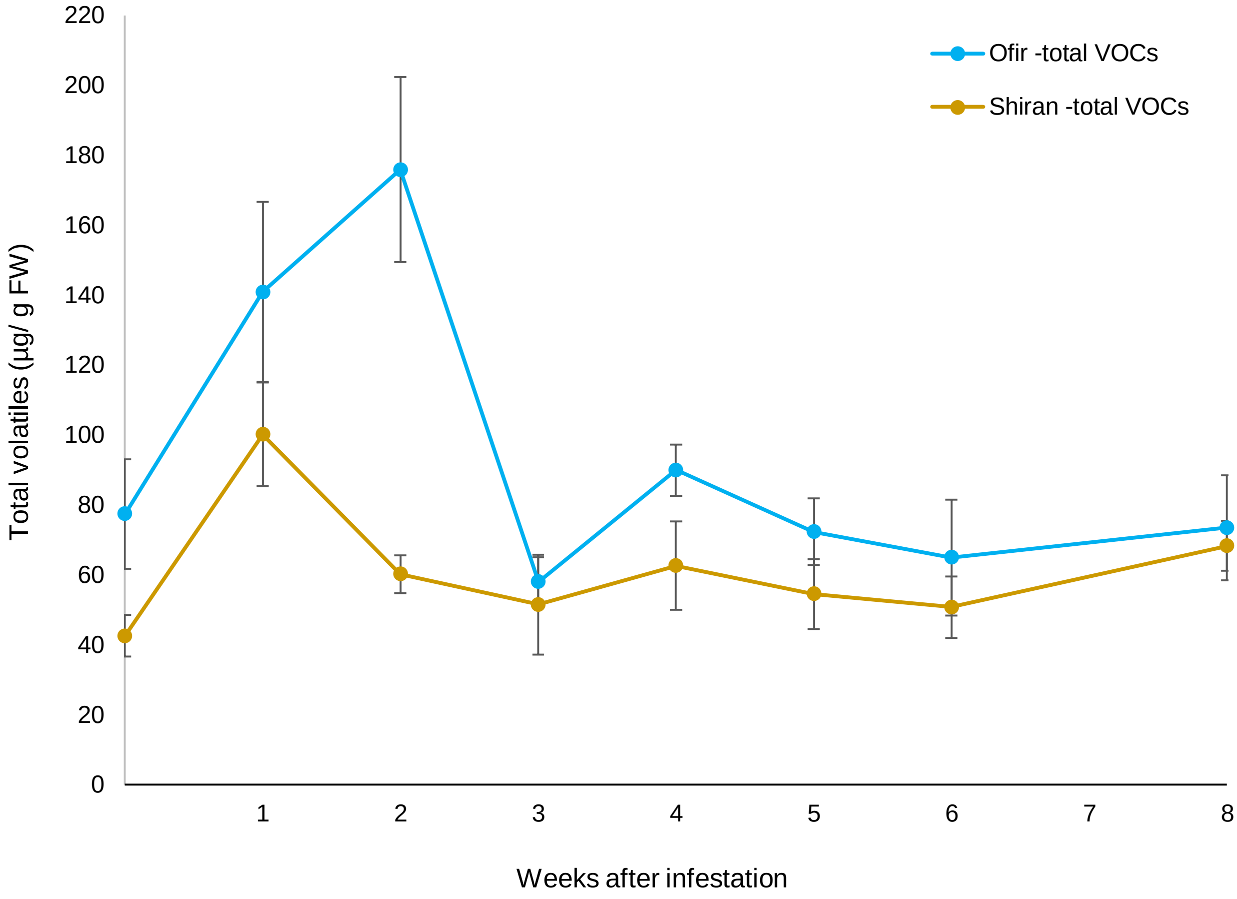

Figure S7. The total levels of VOCs of the two tomato cultivars Ofir and Shiran grown in a net house for three months. TSSMs were introduced during Aug. n = 4-5.

Figure S8

A)

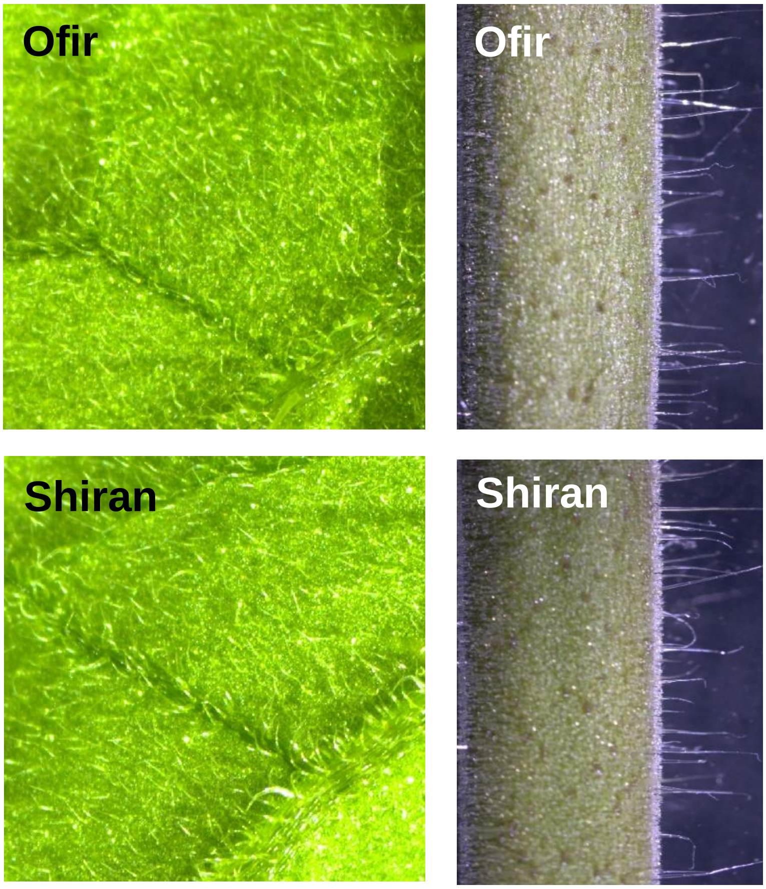

B)

Tissue	Mean	SE
Ofir leaf	200.3	2.2
Ofir stem	27.5	0.9
Shiran leaf	198.5	1.7
Shiran stem	25.8	1.1

Figure S8. Morphology and density of trichomes of Ofir and Shiran cultivar, leaf, and stem. (A) Photos of trichomes of abaxial leaf and stem (B) Summary table of trichomes counted from four leaf disk (1 cm^2) and four stems (1 cm long). Five-week-old plants were used.
